# Cerebral cortical alterations in adolescent early-onset psychosis: a surface-based morphometry mega-analysis

**DOI:** 10.1038/s41380-026-03641-0

**Published:** 2026-05-19

**Authors:** Stener Nerland, Claudia Barth, Kjetil Nordbø Jørgensen, Laura A. Wortinger, Josefina Castro-Fornieles, Covadonga M. Díaz-Caneja, Joost Janssen, Celso Arango, Gisela Sugranyes, Inmaculada Baeza, Fabrizio Piras, Nerisa Banaj, Federica Piras, Simone Elia, Daniela Vecchio, Mathias Lundberg, Hannes Bohman, Bjørn Rishovd Rund, Elena de la Serna, Adriana Fortea, Christian K. Tamnes, Lars T. Westlye, Runar E. Smelror, Dimitrios Andreou, Erik G. Jönsson, Matthew J. Kempton, Shuqing Si, Marinos Kyriakopoulos, Carrie E. Bearden, Kristen Laulette, Saül Pascual-Diaz, Anne-Kathrin J. Fett, Katherine H. Karlsgodt, Lydia Krabbendam, Peter Kochunov, Sophia I. Thomopoulos, Neda Jahanshad, Anthony James, Sophia Frangou, Anne M. Myhre, Nils Eiel Steen, Ole A. Andreassen, Paul M. Thompson, Ingrid Agartz

**Affiliations:** 1 Division of Mental Health and Substance Abuse, Diakonhjemmet Hospital, Oslo, Norway; 2 Division of Mental Health and Addiction, Institute of Clinical Medicine, University of Oslo, Oslo, Norway; 3 Department of Psychiatry and Neurosciences (CCM), Charité – Universitätsmedizin Berlin, Berlin, Germany; 4 Research Unit Gender in Medicine, Charité – Universitätsmedizin Berlin, Berlin, Germany; 5 Division of Mental Health and Addiction, Vestre Viken Hospital Trust, Drammen, Norway; 6 Department of Psychology, Oslo New University College, Oslo, Norway; 7 Department of Child and Adolescent Psychiatry and Psychology, Hospital Clínic of Barcelona, IDIBAPS, CIBERSAM. Department of Medicine, Institute of Neuroscience, University of Barcelona, Barcelona, Spain; 8 Department of Child and Adolescent Psychiatry, Institute of Psychiatry and Mental Health, Hospital General Universitario Gregorio Marañón, IiSGM, CIBERSAM, ISCIII, Madrid, Spain; 9 School of Medicine, Universidad Complutense, Madrid, Spain; 10 Hospital Universitario La Paz, IdiPAZ, School of Medicine, Universidad Autónoma de Madrid, CIBERSAM, Madrid, Spain; 11 Department of Child and Adolescent Psychiatry and Psychology, Hospital Clínic of Barcelona, IDIBAPS, CIBERSAM, Barcelona, Spain; 12 Laboratory of Neuropsychiatry, Department of Clinical Neuroscience and Neurorehabilitation, Santa Lucia Foundation IRCCS, Rome, Italy; 13 Department of Clinical Science and Education Södersjukhuset, Karolinska Institutet, Stockholm, Sweden; 14 Department of Neuroscience, Child and Adolescent Psychiatry, Uppsala University, Uppsala, Sweden; 15 Department of Psychology, University of Oslo, Oslo, Norway; 16 Institut d’Investigacions Biomèdiques August Pi I Sunyer (IDIBAPS), Barcelona, Spain; 17 Centro de Investigación Biomédica en Red de Salud Mental (CIBERSAM), Instituto de Salud Carlos III, Madrid, Spain; 18 Adult Psychiatry and Psychology Department, Institute of Neurosciences, Hospital Clínic de Barcelona, Barcelona, Spain; 19 PROMENTA Research Centre, Department of Psychology, University of Oslo, Oslo, Norway; 20 KG Jebsen Centre for Neurodevelopmental Disorders, University of Oslo, Oslo, Norway; 21 Centre for Precision Psychiatry, Division of Mental Health and Addiction, Oslo University Hospital, Oslo, Norway; 22 Centre for Psychiatry Research, Department of Clinical Neuroscience, Karolinska Institutet & Stockholm Health Care Services, Stockholm Region, Stockholm, Sweden; 23 Department of Psychosis Studies, Institute of Psychiatry, Psychology & Neuroscience, King’s College London, London, United Kingdom; 24 1st Department of Psychiatry, Eginition Hospital, National and Kapodistrian University of Athens, Athens, Greece; 25 Department of Child and Adolescent Psychiatry, Institute of Psychiatry Psychology and Neuroscience, King’s College London, London, UK; 26 South London and Maudsley NHS Foundation Trust, London, UK; 27 Department of Psychiatry and Biobehavioral Sciences, Semel Institute for Neuroscience and Human Behavior, UCLA, Los Angeles, California USA; 28 Department of Psychology, UCLA, Los Angeles, California USA; 29 Magnetic Resonance Imaging Core Facility, August Pi i Sunyer Biomedical Research Institute (IDIBAPS), University of Barcelona, Barcelona, Spain; 30 Department of Psychological Medicine, Institute of Psychiatry, Psychology & Neuroscience, King’s College London, London, UK; 31 Department of Psychology and Neuroscience, School of Health and Medical Sciences, City St George’s University of London, London, UK; 32 Department of Psychiatry and Biobehavioral Sciences, UCLA, Los Angeles, California USA; 33 Department of Clinical, Neuro, and Developmental Psychology, Institute for Brain and Behavior Amsterdam, Vrije Universiteit Amsterdam, Amsterdam, the Netherlands; 34 Department of Psychiatry and Behavioral Sciences, The University of Texas Health Science Center Houston, Houston, TX USA; 35 Imaging Genetics Center, Mark & Mary Stevens Neuroimaging & Informatics Institute, Keck School of Medicine, University of Southern California, Marina del Rey, CA USA; 36 Department of Psychiatry, University of Oxford, Oxford, UK; 37 Highfield Unit, Warneford Hospital, Oxford, UK; 38 Department of Psychiatry, Icahn School of Medicine at Mount Sinai, New York, New York, USA; 39 Section of Child and Adolescent Mental Health Research, Division of Mental Health and Addiction, Oslo University Hospital, Oslo, Norway; 40 Division of Mental Health and Addiction, Oslo University Hospital, Oslo, Norway

**Keywords:** Diseases, Neuroscience

## Abstract

Cortical brain morphology in early-onset psychosis (EOP; age of onset <19 years) is poorly understood, partly due to recruitment constraints linked to its low incidence. We pooled T1-weighted magnetic resonance imaging (MRI) data from 387 adolescents with EOP (mean age=16.1 ± 1.5; 49.6% female) and 338 healthy controls (CTR; mean age=15.8 ± 1.9, 54.4% female) from nine research sites worldwide. Using harmonized processing protocols, we extracted cortical brain metrics from 34 bilateral regions. Univariate regression analyses revealed widespread lower bilateral cortical thickness (left/right hemisphere: *d* = −0.36/−0.31), surface area (left/right: *d* = −0.42/−0.41), cortical volume (left/right: *d* = −0.58/−0.56), and Local Gyrification Index (LGI; left/right: *d* = −0.39/−0.52) in EOP relative to CTR. Diagnostic subgroup analyses showed broader and more pronounced case-control differences in early-onset schizophrenia for area, volume, and LGI. We found no associations with antipsychotic medication use, illness duration, age of onset, or positive symptoms. Negative symptoms were related to smaller left lingual volume (partial *r* = −0.21; *p*_*FDR*_ = 0.014) and antidepressant users had smaller area (*d* = −0.43; *p*_*FDR*_ = 0.034) and volume (*d* = −0.50; *p*_*FDR*_ = 0.003) of the right rostral anterior cingulate compared to non-users. Cortical thickness alterations in EOP showed a similar pattern to those observed in prior studies on adults with schizophrenia (SCZ; *r* = 0.62) and bipolar disorders (BD; *r* = 0.61). However, surface area alterations were overall 1.5 times greater for EOP than adult SCZ and 4.6 times greater than adult BD. In the largest study of its kind, we observed a widespread pattern of cortical alterations in adolescents with psychotic disorders, highlighting the potential impact of aberrant neurodevelopment on cortical morphology in this clinical group.

## Introduction

The morphology of the cerebral cortex has been extensively studied in adults with psychotic disorders [1, 2]. Less is known about morphological brain alterations in adolescents with early-onset psychosis (EOP), i.e., psychotic disorders diagnosed before 19 years of age [3]. This knowledge gap is compounded by the relatively low incidence of EOP (~ 0.6%), making the recruitment of large samples challenging and limiting the statistical sensitivity and robustness required for state-of-the-art neuroimaging analyses. Moreover, EOP encompasses a heterogeneous clinical spectrum, including early-onset schizophrenia (EOS; persistent psychotic symptoms), affective psychosis (AFP; psychosis during mood episodes, typically mood-congruent) and other non-affective psychotic disorders (OTP; non-affective, variable presentation) [4]. This clinical heterogeneity necessitates large sample sizes to enable subgroup analyses. We therefore formed the EOP Working Group within the Enhancing NeuroImaging Genetics Through Meta Analysis consortium (ENIGMA; http://enigma.ini.usc.edu) [5] to facilitate international collaboration between research groups collecting magnetic resonance imaging (MRI) and clinical data on adolescents with EOP.

Psychotic disorders most commonly emerge in early adulthood [6, 7], with an estimated lifetime prevalence of approximately 3% [8]. The incidence during adolescence is lower, with one Danish nationwide population-based cohort study estimating a cumulative incidence of 0.76% in girls and 0.48% in boys before the age of 18 [9]. Despite its rarity, EOP is a leading contributor to the global disease burden [10], driven by its prolonged course, severe and lasting functional impairment, and disruption of critical social and educational milestones during a critical developmental period [11]. Earlier psychosis onset is associated with worse long-term outcomes [12], with EOS linked to a poorer prognosis than adult-onset schizophrenia (SCZ) [13]. Psychosis onset during adolescence coincides with critical brain maturational processes, hypothesized to heighten vulnerability to structural and functional brain alterations [14] and to reflect greater neurodevelopmental involvement than in adult-onset SCZ [14, 15]. Specifically, EOS may exhibit more pronounced neuroanatomical alterations, reflecting greater illness severity and developmental and functional impairments than AFP and OTP [16, 17]. There is an urgent need to advance our understanding of cortical brain alterations in EOP, their associations with clinical characteristics, and how they compare with those observed in adults with SCZ.

Prior MRI studies have reported lower cortical thickness, surface area, and cerebral grey matter (GM) volume in children and adolescents with psychotic disorders compared to healthy controls [14, 15, 18–22]. In an ENIGMA-EOP voxel-based morphometry (VBM) study, we detected lower regional GM volumes across most of the cerebral cortex [23], which were most pronounced in the cingulate and the frontal and temporal cortices. Furthermore, lower volumes in the cerebellum, thalamus, and left inferior parietal gyrus were associated with older age of onset. These findings are consistent with prior studies of adolescents with EOP, although alterations were greater than those reported in another meta-analysis of brain abnormalities in EOS [24]. Early longitudinal studies of childhood-onset schizophrenia (COS; onset <12 years) have reported a fourfold greater decline in cortical GM volume [25], as well as accelerated GM volume loss progressing from parietal to temporal and prefrontal regions [26], in COS compared with healthy controls. More recently, Pina-Camacho et al. [27] reported accelerated longitudinal cortical thinning over a two-year follow-up in adolescent-onset (mean age 17 years), but not adult-onset (mean age 26.5 years), patients with first-episode psychosis [27], indicating more pronounced longitudinal cortical thinning in adolescents with EOP [28].

Most previous cortical studies in EOP have employed VBM, whereas fewer have used surface-based morphometry (SBM) [18, 29, 30], which may offer enhanced sensitivity to pathological alterations in certain clinical conditions [31] and tissue types, notably the cerebral cortex [32]. SBM also distinguishes cortical thickness and surface area, two imaging phenotypes that contribute to cortical GM volume but reflect partly independent genetic, neurobiological, and maturational processes [33–37]. In the present study, we used an SBM approach based on FreeSurfer [38, 39], enabling direct comparisons with prior large-scale ENIGMA studies of cortical thickness and surface area in adults with SCZ [40] and bipolar disorders (BD) [41]. Moreover, SBM allows quantification of cortical folding using the Local Gyrification Index (LGI) [42]. Although both surface area and LGI relate to early neurodevelopmental processes, they capture complementary features: surface area reflects early arealization and tangential expansion, and LGI indexes cortical folding largely established prenatally [43]. Considering both metrics therefore allows detection of alterations in cortical expansion, folding, or both, processes of particular interest in disorders with a known or suspected neurodevelopmental aetiology. Prior studies in adolescent psychosis reported alterations in surface area [15] and cortical folding [44, 45]; however, small sample sizes and inconsistent folding metrics limit the generalizability of prior findings.

Here, we conducted a large-scale mega-analysis of cortical thickness, surface area, cortical volume, and LGI in 387 adolescents with EOP and 338 age-matched healthy controls from nine research sites worldwide. Drawing on prior adolescent and adult studies of psychosis, we hypothesized lower thickness, particularly in the cingulate, temporal, and frontal cortices, smaller global surface area, and lower LGI, particularly in the left precentral gyrus, right middle temporal gyrus, and the right precuneus [46]. In diagnostic subgroup analyses, we hypothesized greater cortical alterations in EOS than AFP or OTP. To probe the hemispheric specificity of case-control differences, we investigated the cortical asymmetry index [47, 48]. We further compared the pattern of cortical morphological alterations in adolescents with those of adults with SCZ and BD, and hypothesized that surface area and LGI would be more severely affected in adolescents with EOP. For all cortical metrics, we assessed sex differences and the impact of antipsychotic and antidepressant medication use, psychotic symptom severity, age of onset, and duration of illness. Given the paucity of studies of cortical morphology in EOP, these analyses were exploratory in nature. Finally, to assess cross-site heterogeneity, we conducted a supplementary meta-analysis.

## Materials & methods

### Study sample

The mega-analysis dataset comprised 387 individuals with EOP (49.4% female) and 338 healthy controls (CTR; 54.4% female) recruited at nine research sites participating through the ENIGMA-EOP Working Group (site overview in Table S1). Among patients, 57.6% had early-onset schizophrenia (EOS; *n* = 223), 26.9% had affective psychoses (AFP; *n* = 104), and 15.5% had other psychotic disorders (OTP; *n* = 60). Site-wise recruitment procedures are described in Table S2. Symptoms were assessed with the Positive and Negative Syndrome Scale (PANSS) [49] or the Scale for the Assessment of Negative Symptoms (SANS) [50] and the Scale for the Assessment of Positive Symptoms (SAPS) [51]. Antipsychotic medication dose was converted to chlorpromazine-equivalent doses (CPZ) [52]. Demographic and clinical information and group differences for the mega-analysis dataset are presented in Table 1. Missing variables are reported in Note S1. Demographic and clinical information stratified by site is given in Table S3 and site-wise definitions of age at onset and duration of illness are reported in Table S4. One additional dataset (*n* = 20 EOP; *n* = 25 CTR) was included only in the supplementary meta-analysis and is described separately in Table S5. All study participants and/or their legal guardians provided written informed consent with approval from local institutional review boards and respective ethics committees. The study was conducted in accordance with the Declaration of Helsinki.

**Table 1:** Demographic and clinical information.

	CTR (*N* = 338)	EOP (*N* = 387)	EOS (*N* = 223)	AFP (*N* = 104)	OTP (*N* = 60)	Test of difference
Age [years]	15.8 ± 1.9	16.1 ± 1.5	16.2 ± 1.6	16.2 ± 1.4	15.8 ± 1.5	F = 7.98, *p* = 0.0049 EOP > CTR
Sex [female]	184 (54.4%)	191 (49.4%)	94 (42.2%)	62 (59.6%)	35 (58.3%)	N.S.
Parental education [years]	13.5 ± 4.1	11.8 ± 4.2	11.2 ± 4.8	12.0 ± 3.4	13.8 ± 2.6	F = 17.21, *p* = 4.04×10^−5^ CTR > EOP
Handedness [right]	233 (88.9%)	221 (88.8%)	155 (90.1%)	32 (82.1%)	34 (89.5%)	N.S.
IQ	106.3 ± 13.3	89.9 ± 17.2	87.5 ± 17.5	93.1 ± 17.4	95.9 ± 13.7	F = 163.80, *p* = 2×10^−16^ CTR > EOP
Age at onset [years]	N.A.	15.2 ± 1.9	15.0 ± 2.0	15.7 ± 1.5	14.9 ± 1.9	F = 6.18, *p* = 0.0023 AFP > EOS, OTP
Duration of illness [years]	N.A.	1.0 ± 1.2	1.2 ± 1.4	0.5 ± 0.7	1.0 ± 1.2	F = 12.78, *p* = 4.36×10^−6^ EOS, OTP > AFP
PANSS Positive	N.A.	19.7 ± 6.8	20.0 ± 6.5	20.8 ± 7.3	17.0 ± 6.3	F = 4.55, *p* = 0.0113 EOS, AFP > OTP
PANSS Negative	N.A.	16.7 ± 7.0	17.9 ± 6.9	14.8 ± 6.7	14.1 ± 6.1	F = 8.98, *p* = 0.0002 EOS > AFP, OTP
SAPS	N.A.	19.2 ± 13.4	17.8 ± 13.2	25.4 ± 12.6	18.4 ± 14.8	N.S.
SANS	N.A.	29.0 ± 17.2	28.4 ± 17.8	27.7 ± 16.0	31.8 ± 18	N.S.
AP use [yes]	N.A.	301 (87.5%)	173 (87.8%)	86 (88.7%)	42 (84.0%)	N.S.
CPZ [mg/day]	N.A.	241.9 ± 204.4	269.1 ± 222.7	215.6 ± 172.4	196.2 ± 180.4	F = 3.49, *p* = 0.0318 EOS > AFP, OTP
Antiepileptic use [yes]	N.A.	13 (4.1%)	7 (3.6%)	6 (7.1%)	0 (0%)	N.S.
Antidepressant use [yes]	N.A.	74 (26.1%)	27 (17.1%)	40 (47.6%)	7 (17.1%)	*χ*^2^ = 24.03, 9.69 AFP > EOS, OTP
Lithium use [yes]	N.A.	26 (8.6%)	3 (1.6%)	23 (27.4%)	0 (0%)	*χ*^2^ = 40.76, 10.49 AFP > EOS, OTP

Sample description for the mega-analysis dataset. Continuous variables are given as means ± standard deviations and categorical variables as counts (percentage of non-missing). Group differences were assessed using Analysis of Variance (ANOVA) for continuous and *χ*^2^ tests for categorical variables. Comparisons were made between EOP and CTR for demographics and IQ, and among EOS, AFP, and OTP for clinical variables. AFP > EOS, OTP. *PANSS* positive and negative syndrome scale, *SAPS* scale for the assessment of positive symptoms, *SANS* scale for the assessment of negative symptoms, *AP* antipsychotic medication, *CPZ* chlorpromazine-equivalent antipsychotic medication dose, *N.A.* not applicable, *N.S.* not significant.

### MRI acquisition and processing

T1-weighted brain images (acquisition parameters in Table S6) were processed with the *recon-all* pipeline of FreeSurfer (v7.1.0 or above) [38, 39] to extract mean cortical thickness, surface area, cortical volume, and LGI for the 68 regions of the Desikan-Killiany atlas (34 per hemisphere; atlas overview in Figure S1). Briefly, the *recon-all* pipeline corrects for intensity nonuniformities, creates cortical surface meshes representing the cortex/non-brain and grey/white matter interfaces, and parcellates the cerebral cortex using sulcogyral folding patterns. Intracranial volume (ICV) was computed with Sequence Adaptive Multimodal SEGmentation (SAMSEG) [53], which may be more robust compared to registration-based approaches [54]. We applied harmonized quality control, including visual inspection of T1-weighted images and surface reconstructions (Note S2). To adjust for scanner-related variation, we used ComBat harmonization [55, 56] as described in Note S3.

### Statistical analyses

Statistical analyses were conducted in R (v4.2.4) [57]. For each cortical metric and set of analyses, we controlled the False Discovery Rate (FDR) with the Benjamini-Hochberg procedure [58]. Group differences in cortical metrics were considered statistically significant at *p*_*FDR*_ < 0.05. Cohen’s *d* effect sizes were computed from *t*-values using the *effectsize* package in R. Model specifications and further details are given in Note S4 and a flowchart of the analyses is shown in Figure S2.

### Demographic and clinical group differences

We tested for group differences in age, sex, parental education, handedness, IQ, duration of illness, age at onset, symptom scores, antipsychotic medication use, CPZ-equivalent dose, and antiepileptic and antidepressant use. We compared EOP and CTR for demographic variables and IQ, and EOS, AFP, and OTP for clinical variables. Continuous variables were assessed using Analysis of Variance (ANOVA), with pairwise *t*-tests to determine the direction of significant differences, and categorical variables were assessed with *χ*^*2*^ tests.

### Case-control differences

To test for case-control differences in cortical metrics, we fitted regression models adjusted for age, age^2^, and sex, with diagnostic group (CTR/EOP) as variable of interest (main model). This model was fitted for each cortical metric and bilateral region, as well as for global cortical metrics, yielding 70 models for each cortical metric. We included age^2^ as a covariate given putative nonlinear aging effects in adolescence [59]. To quantify the hemispheric specificity of case-control differences, we computed the cortical asymmetry index, defined for each cortical region as the difference between measurements in the left and right hemispheres, divided by their mean [47]. We then fitted regression models with the asymmetry indices as outcomes with the same predictors as in the main model. To characterize between-site heterogeneity, we conducted meta-analyses with the *metafor* package in R [60] (Note S6), including an additional dataset (Table S5).

### Diagnostic subgroup analyses

To assess case-control differences for diagnostic subgroups, we used the same model specification as in the main analysis, where the diagnostic group term was replaced with diagnostic subgroup (EOS/AFP/OTP) with CTR as reference. Since diagnostic stability was expected to differ between subgroups, we performed a post-hoc analysis of diagnostic stability (Note S7).

### Regional specificity and adjustment for intracranial volume

To examine the regional specificity of case-control differences, we fitted regression models for each cortical region where the global values of each cortical metric (i.e., mean cortical thickness, total surface area, total volume, and mean LGI) were included as an additional covariate. These models assess regional group differences between EOP and CTR, adjusted for sex, age, and age^2^, relative to the global value of each cortical metric, and capture case-control differences beyond non-region-specific group differences. Since cortical measures may be influenced by head size and a prior ENIGMA-EOP study showed large case-control differences for ICV [61], we conducted an additional sensitivity analysis by fitting separate models adjusted for age, age^2^, sex, and ICV.

### Medication effects and associations with clinical variables

Antipsychotic medication effects were assessed by contrasting patients with EOP using (*n* = 301) with those not using (*n* = 43) antipsychotic medication in separate regression models. Similarly, we contrasted patients using antidepressants (*n* = 74) with non-users (*n* = 208). We further assessed associations between cortical morphology and CPZ among patients using antipsychotic medication (*n* = 268). We tested for associations between cortical morphology and PANSS negative and positive symptom scores, duration of illness, and age of onset by fitting separate regression models for each of these variables adjusted for age, age^2^, and sex. To quantify associations between continuous clinical variables and cortical metrics, we computed partial regression coefficients from the fitted models, adjusting for sex, age, and age^2^, using the *effectsize* package in R.

### Interactions with age and sex and sex-stratified analyses

Interactions of diagnostic group with sex and age were assessed with two separate regression models including interaction terms: 1) age-by-group, adjusted for sex, and 2) sex-by-group, adjusted for age and age^2^. We performed complementary sex-stratified analyses by fitting regression models adjusted for age and age^2^, with diagnostic group (CTR/EOP) as the variable of interest for males and females separately.

### Comparison to adults with schizophrenia and bipolar disorders

To compare case-control differences in EOP with those of adult SCZ and adult/youth BD, we first computed Pearson correlations based on Cohen’s *d* effect sizes from case-control comparisons for EOP, EOS, AFP, and OTP, and the cortical alterations reported in two prior ENIGMA studies [40, 41]. Two-tailed add-one-corrected p-values were estimated using hemisphere-preserving spin permutations (10,000 rotations) with BrainSpace (v0.1.4) [62]. Differences between correlation coefficients were evaluated using Steiger’s test [63]. To assess group differences in the magnitude of effects, we conducted paired *t*-tests across regions and calculated the ratio of the mean regional effect sizes in EOP to that of each comparison group, yielding an index of the average proportional difference in effect size magnitudes across cortical regions.

Cohen’s *d* for case-control differences in cortical thickness and surface area were obtained from an ENIGMA-SZ study of 4474 adults with SCZ [40], with a sample-size weighted mean age of 32.3 years and range of 21.2–43.6 years. For BD, estimates were obtained from an ENIGMA-BD study of 1837 adults and 411 youths with BD ( < 25 years) [41]. Youths with BD had a mean age of 21.1 years and illness onset of 20.3 years. Effect sizes were extracted with the ENIGMA Toolbox (v2.01; https://github.com/MICA-MNI/ENIGMA) [64]. To enable direct comparison with prior studies, we recomputed Cohen’s *d* for the case-control differences between EOP and CTR. For the comparison with SCZ, we adjusted for sex and age but not age^2^. For the comparison with BD, we adjusted for sex and age for cortical thickness, and sex, age, and ICV for surface area. Regional case-control differences were individually compared using *z*-tests as in a previous ENIGMA-EOP study [40]. See Note S5 for further details.

## Results

### Demographic and clinical group differences

Patients with EOP (mean age=16.1 years) were older than CTR (mean age=15.8 years), with similar sex distributions. Parental education and IQ were lower in EOP than in CTR. We found older age of onset and more antidepressant users in AFP relative to EOS and OTP. Duration of illness was higher in EOS and OTP relative to AFP. PANSS positive scores were higher in EOS and AFP relative to OTP, whereas PANSS negative scores were higher in EOS compared to both AFP and OTP. CPZ was higher in EOS compared to both AFP and OTP. Finally, there were more antidepressant and lithium users in AFP compared to EOS and OTP. Group comparisons for the other demographic and clinical variables were not statistically significant. See Table 1 for further details on the group differences.

### Case-control differences

**Fig. 1:**
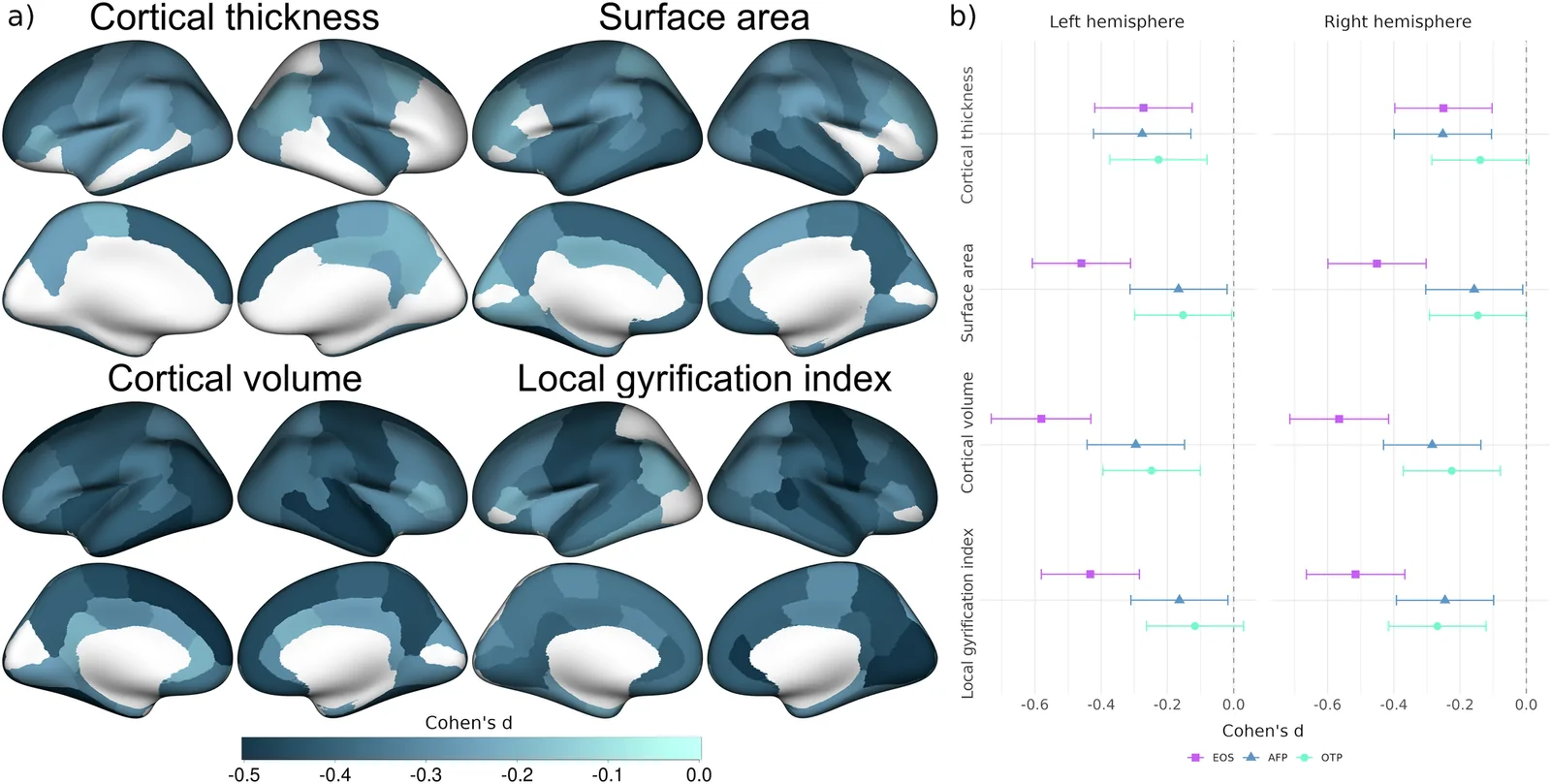
Group differences in cortical morphology in early-onset psychosis (EOP) and diagnostic subgroups. **a** Cohen’s *d* effect sizes for cortical regions that differed significantly after FDR-correction between EOP (n = 387) and healthy controls (n = 338), where darker colour indicates greater magnitude. **b** Cohen’s *d* effect sizes for global cortical metrics stratified by the diagnostic subgroups, early-onset schizophrenia (EOS; n = 223; *purple rectangle*), affective psychosis (AFP; n = 104; *blue triangle*), and other psychoses (OTP; n = 60; *green circle*).

We observed a widespread pattern of lower thickness, area, volume, and LGI in EOP relative to CTR. Cortical thinning was bilateral, with more regions showing lower thickness in the left than in the right hemisphere (left/right: 20/15 regions). The largest group differences were observed in the superior frontal region in both hemispheres (left/right: −0.36/−0.37). Area, volume, and LGI were lower across most cortical regions in EOP compared to CTR, with overall larger effect sizes than those for thickness. Global thickness was lower in EOP compared to CTR in both hemispheres (left/right: −0.36/−0.31). Similarly, global area (left/right: −0.42/−0.40), volume (left/right: −0.58/−0.56), and LGI (left/right: −0.39/−0.52) were lower in EOP compared to CTR. See Fig. 1a for cortical effect size maps for significant differences in the case-control comparison and Figures S3-S6 for bar plots of effect sizes for each cortical metric and hemisphere. See Tables S7-S8 for summaries and model fit for global cortical metrics.

Cortical asymmetry analyses showed a leftward skew in EOP for thickness in the inferior temporal (*d* = −0.26; *p*_*FDR*_ = 0.018) and superior parietal (*d* = −0.24; *p*_*FDR*_ = 0.027) regions and a rightward skew for LGI in the insula (*d* = 0.22; *p*_*FDR*_ = 0.047) and the lateral occipital (*d* = 0.23; *p*_*FDR*_ = 0.047) regions. See Figure S7 for a caterpillar plot of asymmetry differences.

The meta-analysis indicated considerable heterogeneity between individual sites, but the results supported the main findings. See Figure S8 for forest plots with estimated group differences for each site and meta-analytic estimates for the global cortical metrics.

### Diagnostic subgroup analyses

**Fig. 2:**
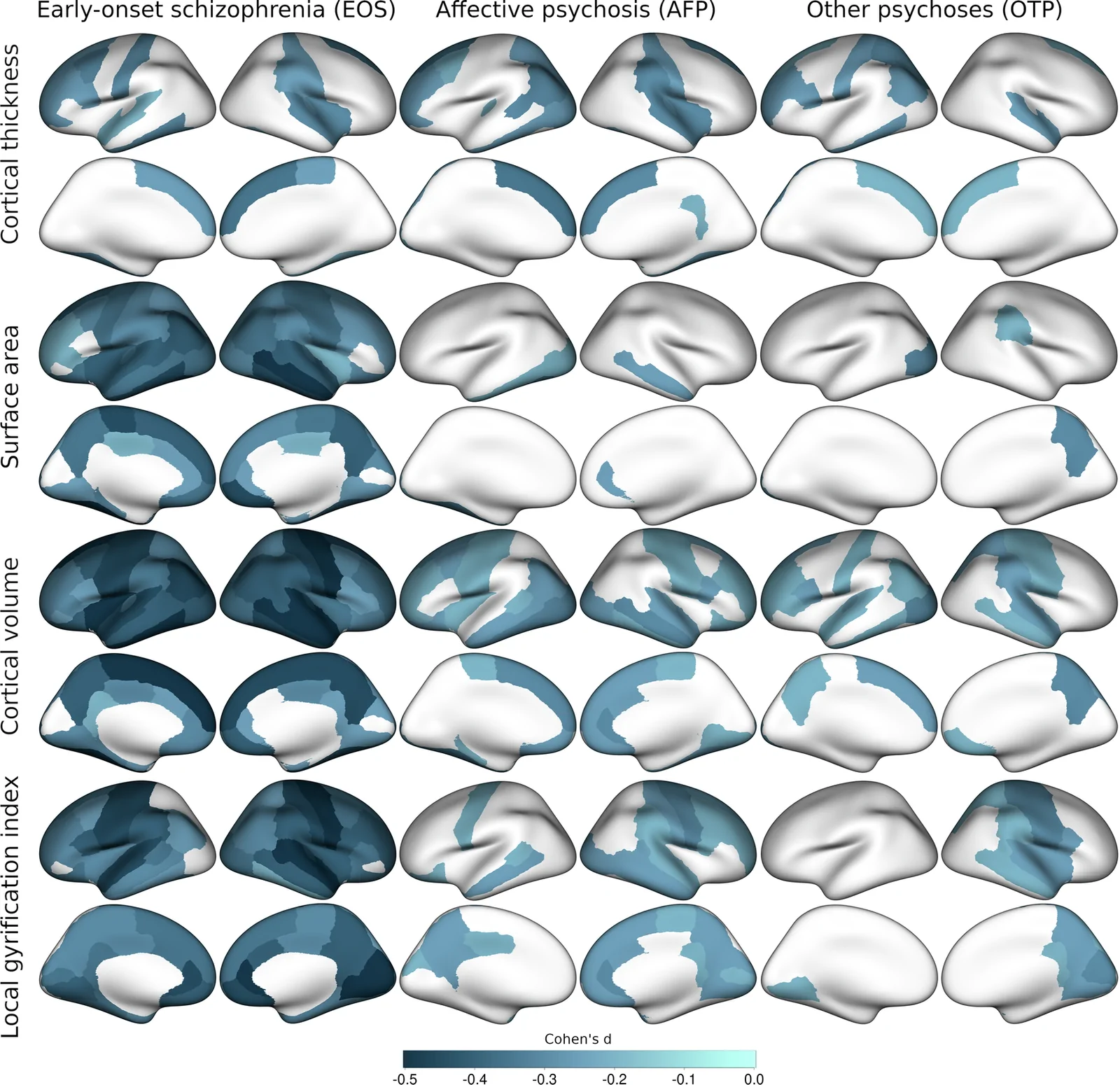
Cohen’s *d* effect sizes for cortical regions that differed significantly between early-onset schizophrenia (EOS; n = 223), affective psychosis (AFP; n = 104), other psychoses (OTP; n = 60), and healthy controls (n=338). Columns correspond to diagnostic subgroups and rows depict statistically significant differences compared to healthy controls for cortical thickness, surface area, cortical volume, and the Local Gyrification Index (LGI).

Effect sizes for global cortical metrics were similar across diagnostic subgroups for thickness, whereas alterations in EOS were greater for area, volume, and LGI (Fig. 1b). We observed a fronto-temporal pattern of cortical thinning in EOS relative to CTR, whereas AFP and OTP had lower thickness in frontal and parietal regions. Area, volume, and LGI were consistently lower in EOS relative to CTR across the cerebral cortex. In contrast, regional surface area only differed significantly for a few cortical regions in AFP (5 regions) and OTP (3 regions) compared to CTR. In both AFP and OTP, lower LGI was mostly seen in the right hemisphere and the cingulate, precuneus, and cuneus for AFP. Only global thickness in the right hemisphere and global LGI of the left hemisphere in OTP did not differ relative to CTR. See Fig. 2 for cortical effect size maps for regions that differed significantly in the diagnostic subgroup analyses. Thickness alterations in EOS had a greater correlation with AFP (*r* = 0.78) than with OTP (*r* = 0.53). Correlations between case-control alterations across diagnostic subgroups are given in Table S9. Post-hoc analysis showed high diagnostic stability for EOS but more moderate stability for AFP and OTP (Note S7).

### Regional specificity and adjustment for intracranial volume

Adjusting for global thickness, we observed higher relative thickness in the left cuneus (*d* = 0.32) in EOP relative to CTR, suggesting this region was relatively preserved despite lower global thickness in EOP. Regional surface area was relatively smaller in the left fusiform gyrus (*d* = −0.28) after adjusting for global surface area. No other case-control comparisons were statistically significant. See Figures S9-S12 for bar plots of effect sizes for each cortical metric and hemisphere adjusted for global cortical metrics.

Adjusting for ICV, case-control differences were similar to the main analyses for thickness and LGI, whereas lateral prefrontal volume differences were attenuated. For area, only differences in the inferior temporal lobe and in medial regions remained significant after ICV-correction. See Figure S13 for cortical effect size maps when adjusting for ICV.

### Medication effects and associations with clinical variables

Cortical metrics did not differ significantly between antipsychotic medication users compared to non-users and there were no significant associations with CPZ. See Tables S10-S11 for model summaries and Figure S14 for uncorrected partial correlations between CPZ and cortical metrics. We observed smaller area (*d* = −0.43; *p*_*FDR*_ = 0.034) and volume (*d* = −0.50; *p*_*FDR*_ = 0.003) in the right rostral anterior cingulate (rACC) in current antidepressant users compared to non-users. For psychotic symptoms, only the association between the PANSS negative subscale and the volume of the left lingual region was statistically significant (partial *r* = −0.21; *p*_*FDR*_ = 0.014). See Figures S15-S16 for partial correlations between the PANSS positive and negative subscales and cortical metrics. There were no associations between cortical morphology and duration of illness or age of onset.

### Interactions with age and sex and sex-stratified analyses

**Fig. 3:**
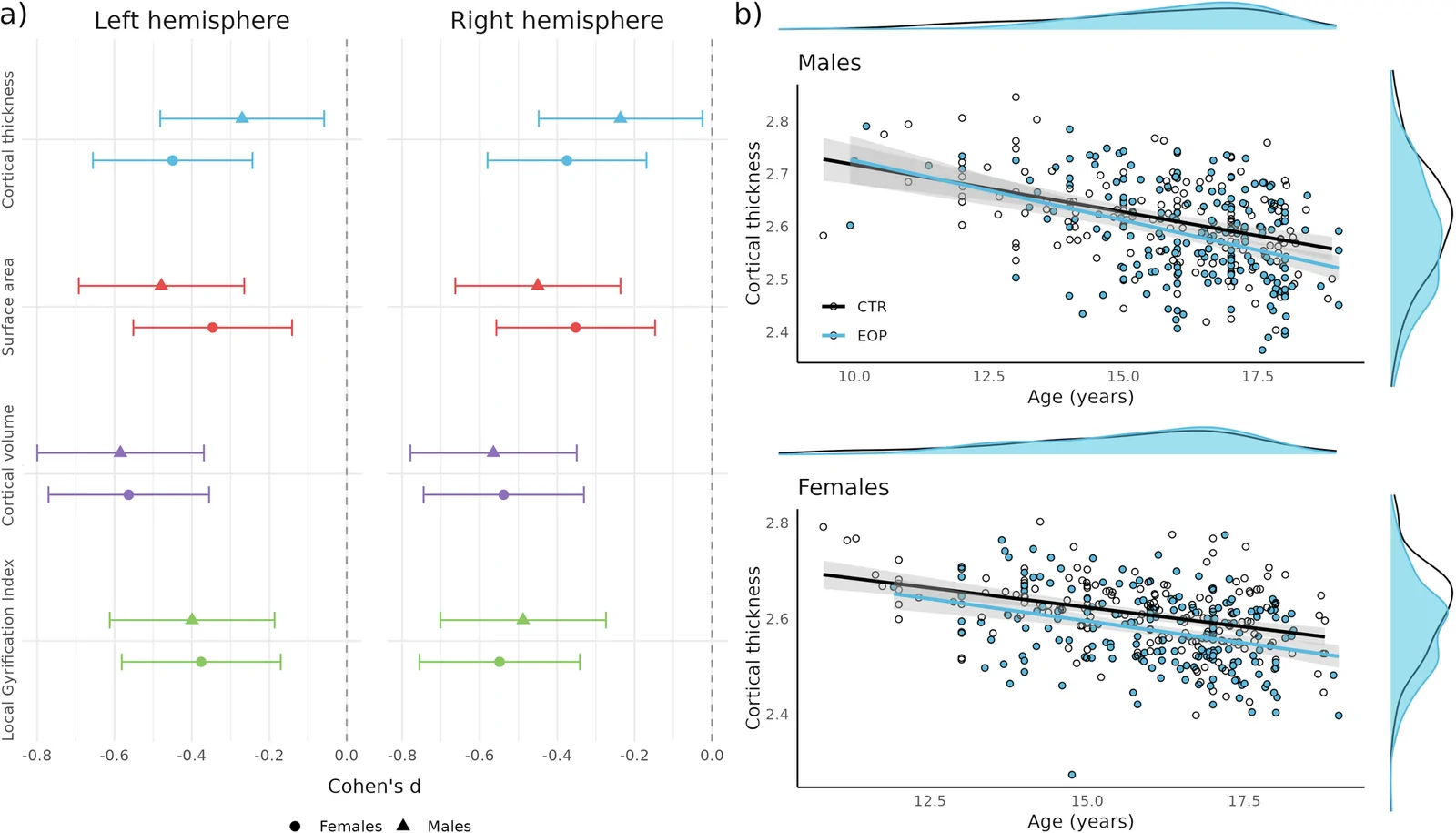
Sex-stratified cortical alterations in early-onset psychosis (EOP). **a** Cohen’s *d* effect sizes for females (n = 191; *circles*) and males (n = 196; *triangles*) with EOP for global cortical thickness, surface area, cortical volume, and Local Gyrification Index (LGI) for each hemisphere. The bars denote 95% confidence intervals. **b** Marginal plots showing global cortical thickness as a function of age stratified by sex with EOP represented with blue and healthy controls with white circles. The blue distributions depict thickness and age distributions for EOP and the black outlines depict the same distributions for same-sex controls (184 females, 154 males).

There were no significant interactions between diagnostic group and age or sex for any cortical metric. Sex-stratified analyses showed lower regional thickness only in female patients compared to female CTR. However, male patients showed global thinning in both hemispheres relative to male CTR. More regions showed smaller area in male than female patients compared to sex-matched CTR, while volume and LGI alterations were similar across sexes. All global cortical metrics differed from those of CTR in both male and female patients (Fig. 3). See Figures S17-S18 for cortical effect size maps and Figures S19–22 for bar plots of effect sizes across all cortical regions from the sex-stratified analyses.

### Comparison to adults with schizophrenia and bipolar disorders

**Fig. 4:**
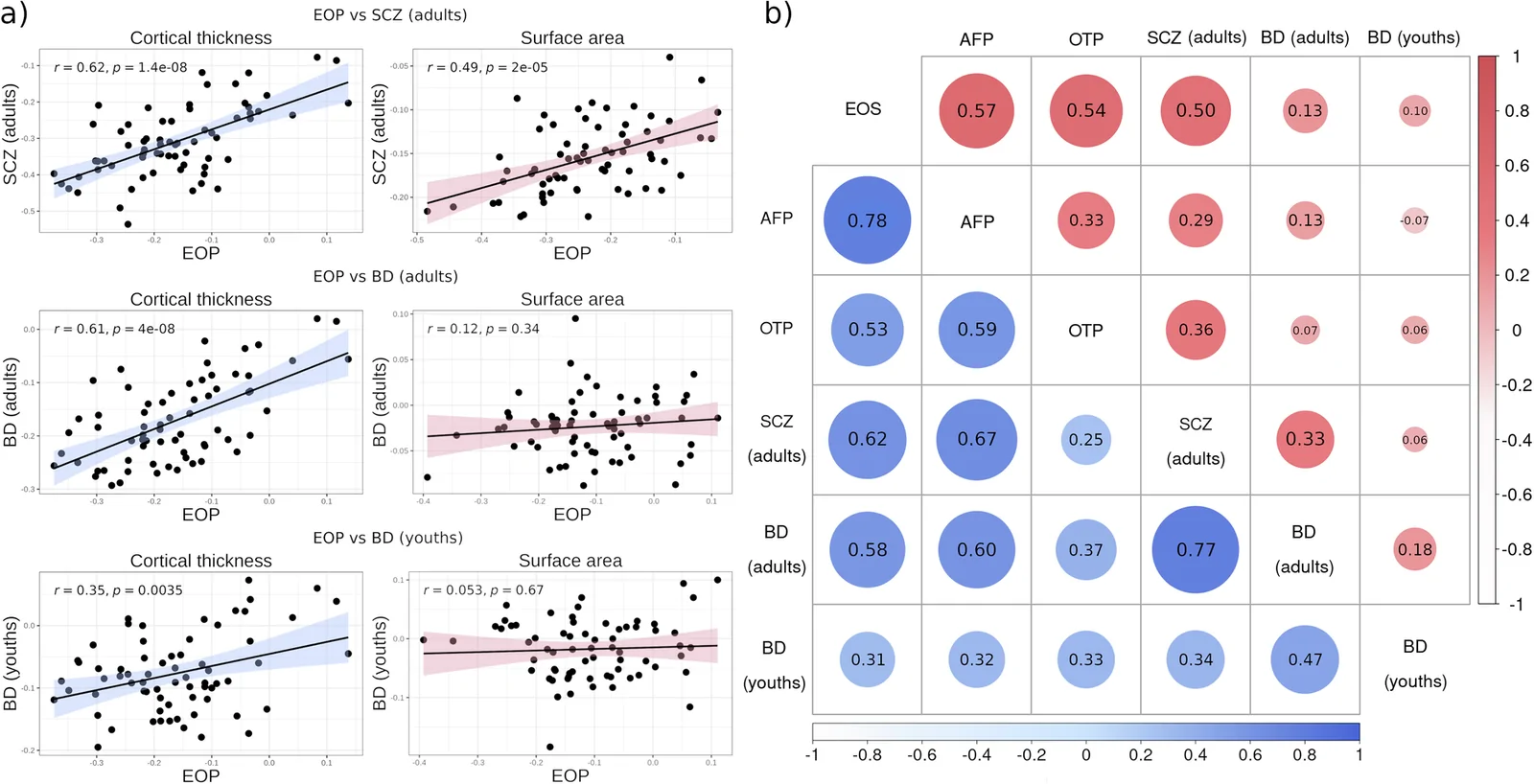
Comparison of cortical alterations across adult schizophrenia (SCZ) and adult/youth bipolar disorder (BD). **a** Correlations between case-control differences in cortical thickness (*blue*) and surface area (*red*) between early-onset psychosis (EOP; n = 387), adults with SCZ (n = 4474) and BD (n = 1837), as well as youths with BD (n = 411). Regional Cohen’s *d* effect sizes for EOP are depicted on the *x*-axes and the *y*-axes represent effect sizes for each comparison group. **b** Correlations between case-control differences for early-onset schizophrenia (EOS; n = 223), affective psychosis (AFP; n = 104), other psychoses (OTP; n = 60), and adults with SCZ and BD, as well as youths with BD. The lower diagonal shows correlations for cortical thickness (*blue*) and the upper diagonal shows correlations for surface area (*red*). Darker colours and larger circles indicate stronger correlations.

We observed strong correlations between regional Cohen’s *d* effect sizes for EOP and adult SCZ for thickness (*r* = 0.62; *p*_*spin*_ = 2.0×10^−4^) and moderate correlations for area (*r* = 0.49; *p*_*spin*_ = 2.0×10^−4^). EOP thickness alterations were more strongly correlated to those of adult BD (*r* = 0.61; *p*_*spin*_ = 2.0×10^−4^) than youth BD (*r* = 0.35; *p*_*spin*_ = 1.6×10^−2^). Surface area alterations in EOP did not correlate significantly with either BD group. In the diagnostic subgroup analyses, we observed strong correlations between cortical thickness alterations in EOS and AFP with those of adult SCZ and adult/youth BD, whereas correlations for cortical thickness alterations in OTP were weak (Fig. 4b).

The magnitudes of Cohen’s *d* effect sizes for cortical thickness were significantly smaller in EOP, EOS, AFP, and OTP compared to adult SCZ (*p*_*FDR*_ < 1.6×10^−16^), but greater compared to youth BD (*p*_*FDR*_ < 9.6×10^−5^). Effect sizes for cortical thickness in EOP and AFP did not differ significantly from those of adult BD but were smaller in EOS (*p*_*FDR*_ = 2.1×10^−3^) and OTP (*p*_*FDR*_ = 6.3×10^−3^). Post hoc analyses indicated that average effect sizes for cortical thickness in EOP were 0.53 times those of adult SCZ and 2.11 times those of youth BD. For surface area, effect sizes were overall greater in EOP, EOS, AFP, and OTP compared to those of adult SCZ (*p*_*FDR*_ < 7.6×10^−4^), adult BD (*p*_*FDR*_ < 1.0×10^−2^), and youth BD (*p*_*FDR*_ < 5.6×10^−3^). Effect sizes for surface area in EOP were 1.51 times those of adult SCZ, 4.62 times those of adult BD, and 6.11 times those of youth BD.

Regional effect size comparisons showed significantly lower effect sizes in EOP for thickness relative to adult SCZ across multiple regions, with more limited differences but greater effect sizes in EOP relative to both adults and youth BD. For area, effect sizes in EOP were greater than for adult SCZ and adult/youth BD. See Figures S23-S25 for Cohen’s *d* effect sizes across EOP and comparison groups and Tables S12-S18 for comparisons of correlations and regional effects.

## Discussion

In the largest study on cortical morphology in adolescent psychosis to date, we found widespread lower cortical thickness, surface area, cortical volume, and LGI in EOP relative to healthy controls. There were more pronounced deficits in surface area, cortical volume, and LGI in EOS than in AFP and OTP. The spatial pattern of cortical thickness alterations in EOP closely resembled those in adult SCZ and BD, whereas surface area alterations correlated only with those of adult SCZ. Moreover, the magnitude of cortical thickness deficits in EOP was approximately half that of adult SCZ, while surface area deficits were about 1.5 times greater in EOP. These findings suggest that cortical thickness alterations may reflect both static and progressive effects, whereas surface area alterations may indicate greater neurodevelopmental involvement in EOP compared to adult-onset SCZ.

A key finding was widespread case-control differences across cortical metrics, suggesting a broad pattern of diffuse alterations. This was supported by the regional specificity analyses where only two regions differed significantly relative to controls when adjusting for global case-control deficits. Similarly, the cortical asymmetry analyses only showed lateralization differences in EOP for three regions relative to healthy controls, indicating largely bilateral case-control differences. The spatial distribution of case-control differences was greatest for cortical volume, LGI and surface area, with fewer significant differences for cortical thickness. The broad cortical alteration pattern is in line with a prior ENIGMA-EOP study on VBM by Si et al. [23], but differed from some smaller studies reporting focal alterations in adolescent psychosis [65, 66]. The greater extent of case-control differences likely reflects higher statistical power, enabling the detection of more subtle brain structural deviations.

EOS showed more widespread and greater differences in surface area, cortical volume, and LGI compared to AFP and OTP. In contrast, global thickness differences were similar across subgroups, although AFP exhibited more significant regional alterations, particularly in the left parietal and occipital lobes. Cortical thickness alterations were strongly correlated between EOS and AFP (*r* = 0.78) and moderately between EOS and OTP (*r* = 0.53). As cortical thickness deficits may reflect both static and progressive processes, more pronounced subgroup differences may arise later in the illness course. The findings partly support the hypothesis that neuroanatomical alterations are more pronounced in EOS. Moreover, larger overall surface area and LGI alterations in EOS are consistent with more substantial neurodevelopmental disruption (areal expansion and gyrification) than in AFP or OTP. However, subgroup sizes differed (57.6% EOS; 26.9% AFP; 15.5% OTP), which influenced statistical sensitivity. Subgroup results should therefore be interpreted alongside effect sizes, which are less biased by unequal subgroup sizes. This highlights the importance of large samples, particularly given the marked clinical heterogeneity in EOP [67].

Diagnostic assessment in children and adolescents can be challenging, as criteria are largely validated in adults and rely on time-based features that may be difficult to ascertain in youth [68]. The stability of diagnoses over time should therefore be considered when interpreting our findings, particularly those related to diagnostic subgroups. In post-hoc analyses on 169 patients with EOP for whom follow-up diagnostic information was available (Note S7), we observed high stability for EOS (95.4%), moderate stability for AFP (63.2%), and the lowest stability for OTP (47.8%). While the EOS estimate aligns closely with prior adolescent and adult studies [69–72], the stability of AFP was somewhat lower than typically reported, likely reflecting small sample sizes and diagnostic heterogeneity. Nonetheless, the pattern is broadly consistent with recent evidence: Pelizza et al. [73] reported 2-year stability rates of 100% for EOS and 75% for AFP [73], while two longer-term studies (mean follow-up 10.5 and 11.5 years) found >80% stability for both EOS and AFP diagnosed in adolescence [71, 72]. At the same time, some diagnoses, notably schizoaffective disorder, may show substantially lower stability [74, 75], and the evidence base in adolescents remains limited. In light of this, our diagnostic subgroup analyses should be interpreted cautiously, especially for OTP, which shows the greatest propensity to transition to EOS or AFP, or remit from psychosis [69, 72, 73].

Longitudinal studies have reported accelerated cortical GM loss, notably in the frontal lobe, in EOP compared to healthy controls [25–27, 76, 77]. Such progressive cortical alterations could explain the smaller effect sizes in EOP relative to adult SCZ. If cortical thinning is progressive, alterations in adolescents with psychosis may eventually resemble the patterns seen in adults, potentially reflecting accelerated synaptic pruning during adolescence [78, 79]. Brain connectivity has been proposed to shape the spatial distribution of cortical thinning in SCZ, with mutually atrophic effects driving coordinated thinning across distant regions [80], supported by studies linking cortical thinning to functional and structural cortico-cortical connectivity [81–85]. However, the role of connectivity in driving these alterations remains unclear, as are the underlying neurobiological mechanisms, which may be confounded by antipsychotic use, lifestyle, adversity, and other exogenous factors. Future research should study the progressive aspects of brain structure in EOP, how connectivity relates to cortical alterations, and whether these changes reflect psychosis-specific effects or broader neurodevelopmental vulnerabilities [86–88].

Compared to previous ENIGMA studies [40, 41], we found that surface area alterations in EOP correlated significantly with those of adult SCZ, but not BD, with the strongest correlations for EOP (*r* = 0.49) and EOS (*r* = 0.50). Importantly, we observed greater surface area alterations in EOP than in adult SCZ. While cortical thinning may be, at least partly, progressive, surface area and LGI stabilize in early adolescence and are considered more closely linked to neurodevelopmental processes [61, 89, 90]. These findings align with the ENIGMA-EOP study on intracranial and subcortical volumes in a partially overlapping sample by Gurholt et al. (2022), which reported the largest case-control effect size for ICV [61], which was greater (*d* = −0.39) than for adult SCZ (*d* = −0.12) [91]. In the present study, case-control differences in surface area were attenuated when adjusting for ICV, and only one region, the left fusiform area, remained significant after adjusting for global surface area. This suggests that surface area alterations are global, may reflect pre-existing brain structural differences, are more specific to psychotic disorders, and may indicate a greater neurodevelopmental burden in EOP than in adult-onset SCZ.

Although sex-by-group interactions were not significant, stratified analyses revealed significant regional cortical thinning only in female patients relative to same-sex healthy controls. This contrasts with findings from the ENIGMA-EOP study by Barth et al. [3], where male patients exhibited greater abnormalities of white matter microstructure than female patients [92]. Nevertheless, both sexes showed significant global cortical thinning relative to controls. Thus, cortical thinning in male patients was less pronounced than in female patients relative to their same-sex counterparts. Adolescence is a critical period of brain maturation, marked by substantial sex differences [93–95]. If cortical thinning reflects progressive pathology, earlier maturation in female patients may be linked to earlier and more pronounced cortical thinning. Over time, both sexes may converge toward the cortical thinning pattern seen in adults with SCZ [80]. Future studies should examine sex-specific mechanisms in EOP, ideally incorporating hormonal and pubertal markers.

We found no significant cortical associations with PANSS positive scores, antipsychotic use or dose, or age-by-group interactions. However, PANSS negative scores were associated with lower left lingual volume, a region involved in visual processing. Prior work in adults with psychotic disorders has linked perceptual deficits to negative symptoms and surface area alterations of the visual cortex have been reported in first-degree relatives, suggesting links to inherited liability [96, 97]. Antidepressant users had significantly lower surface area and volume in the right rACC compared to non-users. The rACC has been implicated in depression and treatment response in Major Depressive Disorder [98–100], thus this finding may reflect comorbid depressive symptoms, which are common in EOP [101]. In contrast to Si et al. [23], we found no association between cortical volume and antipsychotic dose or age of onset. Given strong correlations between age of onset and age (*r* = 0.74), and potential site-specific recruitment biases, disentangling these effects is challenging. Similarly, illness onset and cumulative medication exposure are closely linked, complicating causal inference. Longitudinal studies, especially in at-risk individuals, are needed to clarify how cortical morphology relates to medication exposure, illness duration, and age of onset.

Some important limitations apply. The study design does not permit causal inference, notably regarding medication effects and psychotic symptom severity. A family history of psychosis has been linked to earlier onset, symptom expression, and cognitive deficits [102] and may contribute to the prominence of surface area and LGI alterations in EOP, but was not considered in the present study due to missing data. Hence, future studies with harmonized assessments are needed. We lacked information on pubertal staging, which should be considered in future studies on adolescents. Despite using broad subgroup definitions, residual diagnostic instability may limit the clinical specificity of these analyses, especially for the OTP group [69, 73]. Age and duration of illness are linked, making it difficult to statistically separate their effects. While we reported on parental education as a proxy for socioeconomic status (available for 59.6% of participants), future studies should incorporate richer, multi-domain indices of socioeconomic status. Data were collected from multiple research sites and MRI scanners, introducing both clinical and technical variability, e.g., heterogeneity related to clinical assessments, definitions of age at onset and duration of illness, and scanner-related variation. While this may have introduced noise, it also provided an opportunity to assess between-site variability. In line with recommendations, we applied the scanner harmonization method ComBat, which attenuates scanner-related differences, however residual effects may remain. Similarly, variation in inclusion/exclusion criteria across sites can lead to differences in diagnostic distributions, age ranges, or illness duration and severity, which is difficult to address statistically and represents an important limitation of multi-site studies.

### Conclusions

In the largest study of cortical morphology in adolescents with EOP to date, we found widespread lower cortical thickness, surface area, cortical volume, and LGI. Alterations were most pronounced in EOS and were independent of medication use, illness duration, or symptom severity, except for limited associations with negative symptoms and antidepressant use. The pattern of cortical thickness alterations closely resembled that seen in adults with SCZ and BD. In contrast, the pattern of surface area alterations only correlated with those of adults with SCZ, where the magnitude of the effects was, on average, 1.5 times greater in EOP than in adults with SCZ. The findings suggest that greater surface area alterations may serve as a neurobiological signature of early-onset psychosis, and the larger effect sizes for surface area and LGI in adolescent psychosis highlight the potential impact of aberrant neurodevelopment on cortical morphology in this clinical group.

## Supplementary information

Supplementary Materials

## Data Availability

Study results, analysis scripts, and supporting data are available from the corresponding authors upon reasonable request, subject to approval by the principal investigators and completion of applicable ethical approvals and data transfer agreements.
