# Supplementary material for: Cerebral cortical alterations in adolescent early-onset psychosis: a surface-based morphometry mega-analysis
Source: Mol Psychiatry. 2026 May 19;31(10):5574–85. doi: 10.1038/s41380-026-03641-0 (PMC13569444; doi:10.1038/s41380-026-03641-0)
Supplement: Supplementary file 1 [file 41380_2026_3641_MOESM1_ESM.docx]

Supplementary Materials

**Cerebral cortical alterations in adolescent early-onset psychosis:**

**a surface-based morphometry mega-analysis**

**Table of contents**

1. **Supplementary Notes**

   **Note S1.** Further information on clinical assessments.
   **Note S2.** Quality control procedure.
   **Note S3.** Scanner harmonization.
   **Note S4.** Regression model specifications.
   **Note S5.** Comparison to prior ENIGMA studies.
   **Note S6.** Meta-analysis across sites.
   **Note S7.** Post-hoc analysis of diagnostic stability.
2. **Supplementary Figures**

   **Figure S1.** Overview of cortical regions in the Desikan-Killiany atlas.
   **Figure S2.** Flowchart of analyses.
   **Figure S3-S6.** Main analysis - Bar plots of effect sizes.
   **Figure S7.** Asymmetry analyses - Caterpillar plot of asymmetry indices.
   **Figure S8.** Meta-analysis - Forest plots for global cortical metrics.
   **Figure S9-S12.** Regional specificity analyses - Bar plots of effect sizes.
   **Figure S13.** ICV-adjusted analyses - Surface maps of effect sizes.
   **Figure S14.** CPZ - Surface maps with uncorrected partial correlations.
   **Figure S15.** PANSS positive - Surface maps with uncorrected partial correlations.
   **Figure S16.** PANSS negative - Surface maps with uncorrected partial correlations.
   **Figure S17-S22.** Sex-stratified analyses - Surface maps and bar plots stratified by sex.
   **Figure S23-S25.** Effect size comparison with previous ENIGMA studies.
3. **Supplementary Tables

   Table S1.** Overview of participating research sites.
   **Table S2.** Recruitment procedures and criteria for each site.
   **Table S3.** Site-wise demographic and clinical information.
   **Table S4.** Definitions of age at onset and duration of illness for each site.
   **Table S5.** Demographic and clinical information of additional dataset.
   **Table S6.** MRI acquisition parameters for each site.
   **Table S7.** Model summaries for global cortical metrics.
   **Table S8.** Incremental model fit and explained variance for global cortical metrics.
   **Table S9.** Correlations between case-control alterations across diagnostic subgroups.
   **Table S10-S11.** Model summaries for AP use and CPZ effects on global cortical metrics.
   **Table S12-S18.** Comparisons of correlations and effect sizes.
4. **References**

## 1. Supplementary Notes

**Note S1 - Further information on clinical assessments**

Positive and Negative Syndrome Scale (PANSS) [1] symptom scores were available for 306 participants, while the Scale for the Assessment of Negative Symptoms (SANS) [2] and the Scale for the Assessment of Positive Symptoms (SAPS) [3] were available for 42 participants. Therefore the symptom severity analyses were conducted for the PANSS. Age of onset and duration of illness were missing for 26 participants. Information on current antipsychotic medication use status (yes/no) was available for 344 participants, of which 301 (87.5%) used antipsychotic medication at inclusion. Chlorpromazine-equivalent doses (CPZ) [4] of antipsychotic medication at inclusion were available for 306 participants.

**Note S2 - Quality control procedure**

Quality control (QC) was conducted using a dual approach: 1) We identified outliers using the Median Absolute Deviation (MAD) approach [5] implemented in the *routliers* package in R, with a MAD threshold of 4. The MAD is less biased by sample size and the presence of extreme outliers compared to the mean. Outliers flagged for any cortical metric and region were inspected visually to ensure that the surface reconstruction was acceptable. In the event of major errors or poor image quality, the participant was flagged and excluded from all further analyses. 2) We created QC images for each participant comprising coronal and axial views of the original T1-weighted images with Sequence Adaptive Multimodal SEGmentation (SAMSEG) [6] segmentations overlain. These were inspected visually for all participants, and if major segmentation errors were identified, or image quality was deemed to be unacceptable, the participant was excluded from further analyses. In the dataset used in the mega-analyses, we excluded 27 participants due to poor image quality, 2 participants due to processing errors, and 13 participants due to image artifacts, usually related to dental braces or movement in the scanner.

**Note S3 - Scanner harmonization**

To adjust for scanner-related variation, we applied ComBat harmonization for each cortical metric, i.e., cortical thickness, surface area, cortical volume, and the Local Gyrification Index (LGI), as well as segmentation-based total intracranial volume (ICV), separately. ComBat was developed to adjust for batch effects in microarray experiments and has become widely used for adjusting for scanner-related variation in neuroimaging [7–9]. We used empirical Bayes to leverage within-metric variation across all cortical regions of the Desikan-Killiany atlas [10] and specified age, sex, and case-control status as variables of interest. After ComBat harmonization, global hemispheric metrics showed comparable within-group variation between EOP and CTR. Cortical thickness SDs across hemispheres were 0.08-0.09 mm for EOP and 0.08 mm for CTR, surface area SDs were 8,536-8,680 mm^2^ for EOP and 8,153-8,203 mm^2^ for CTR, cortical volume SDs were 26,079-26,608 mm^3^ for EOP and 25,429-25,507 mm^3^ for CTR, and LGI SDs were 0.13 in both groups.

**Note S4 - Regression model specifications**

Regression models were fitted with the *lm* command from the *stats* package in R [11]. To control the False Discovery Rate (FDR), we used the *p.adjust* command from the same package. FDR-correction was performed for each cortical metric separately, e.g., for the main analysis of cortical thickness, we performed a total of 70 statistical tests (68 cortical regions + 2 global hemispheric thickness values). Cohen's *d* effect sizes were computed from *t*-values from the fitted regression models using the *effectsize* package in R.

In the following regression model specifications, DV_ij_ represents the dependent variable, i.e., cortical thickness, surface area, cortical volume, or the Local Gyrification Index (LGI) for the *j*-th cortical region (one of the 34 regions per hemisphere) for the *i*-ith participant. Similarly, the predictors of the models are indexed by participant. The reference group was healthy controls for Dx (levels: EOP/CTR) and FullDx (levels: EOS/AFP/OTP/CTR). The variable of interest for each model is denoted in bold. Model assumptions (including homoscedasticity) were checked using residual diagnostics with no major deviations detected.

Case-control differences

All participants: DV_ij_ ~ Age_i_ + Age_i_^2^ + Sex_i_ + **Dx_i_**

Diagnostic subgroup analyses

All participants: DV_ij_ ~ Age_i_ + Age_i_^2^ + Sex_i_ + **FullDx_i_**

Regional specificity and adjustment for intracranial volume

1. All participants: DV_ij_ ~ Age_i_ + Age_i_^2^ + Sex_i_ + GlobalMetric_i_ + **Dx_i_**
2. All participants: DV_ij_ ~ Age_i_ + Age_i_^2^ + Sex_i_ + ICV_i_ + **Dx_i_**

Medication effects and associations with clinical variables

1. Non-missing patients: DV_ij_ ~ Age_i_ + Age_i_^2^ + Sex_i_ + **AP_i_**
2. Non-missing patients: DV_ij_ ~ Age_i_ + Age_i_^2^ + Sex_i_ + **AD_i_**
3. Non-missing patients: DV_ij_ ~ Age_i_ + Age_i_^2^ + Sex_i_ + **CPZ_i_**
4. Non-missing patients: DV_ij_ ~ Age_i_ + Age_i_^2^ + Sex_i_ + **PANSS-POS_i_**
5. Non-missing patients: DV_ij_ ~ Age_i_ + Age_i_^2^ + Sex_i_ + **PANSS-NEG_i_**
6. Non-missing patients: DV_ij_ ~ Age_i_ + Age_i_^2^ + Sex_i_ + **Duration of illness_i_**
7. Non-missing patients: DV_ij_ ~ Age_i_ + Age_i_^2^ + Sex_i_ + **Age of onset_i_**

Interactions with age and sex and sex-stratified analyses

1. All participants: DV_ij_ ~ Age_i_ + Sex_i_ + Dx_i_ + **Age_i_ × Dx_i_**
2. All participants: DV_ij_ ~ Age_i_ + Age_i_^2^ + Sex_i_ + Dx_i_ + **Sex_i_ × Dx_i_**
3. Male participants: DV_ij_ ~ Age_i_ + Age_i_^2^ + **Dx_i_**
4. Female participants: DV_ij_ ~ Age_i_ + Age_i_^2^ + **Dx_i_**

**Note S5 - Comparison to prior ENIGMA studies**

To harmonize covariate adjustment across case-control comparisons, we recomputed regional effect sizes for EOP adjusted only for age and sex for cortical thickness, and similarly for surface area in the comparison with adult SCZ. For surface area comparisons involving adults and youths with BD, we also adjusted for ICV. This ensured consistency in the selection of covariates across group comparisons and enabled direct comparison of effect sizes. Pearson correlation coefficients were compared using Steiger's test [12].

We used paired *t*-tests to compare Cohen's *d* effect sizes in EOP with those in adult SCZ, adult BD, and youth BD. To further characterize the average proportional difference in effect sizes between groups, we calculated the ratio of the mean Cohen's *d* effect size in EOP to that of each comparison group. This provided a descriptive measure of the average proportional difference in effect size magnitudes across regions.

Finally, we performed additional pairwise comparisons between region-wise Cohen's *d* effect sizes using a similar approach as in a prior ENIGMA-EOP study [13]. Specifically, we performed *z*-tests based on the following formulas:

In equation 1), *d_Group1_* is the estimated Cohen's *d* effect sizes for the reference group, i.e., EOP, and *d_Group2_* is that of the comparison group (adult SCZ, adult BD, or youths with BD), and SE is the standard error of the estimated Cohen’s *d*. In equation 2) the p-value is computed using a two-tailed test where Φ is the standard normal cumulative distribution.

**Note S6 - Meta-analysis across sites**

For the meta-analysis, we first fitted separate linear regression models for each individual research site, adjusted for age, age^2^, and sex, with diagnostic status as the main variable of interest. The models were fitted separately for global cortical thickness, surface area, cortical volume, and the Local Gyrification Index (LGI) in the right and left hemispheres. For sites with data acquired on multiple MRI scanners, we included scanner as a covariate. We then extracted the t-statistics associated with the Dx term, which were converted to Hedges' *g* with the *effectsize* package in R. The Hedges' *g* was used to correct for small-sample bias. We estimated the standard error, SE*_g_*, of the Hedges' *g* with the formula:

The meta-analysis was conducted using a random-effects model with restricted maximum likelihood (REML), implemented in the *rma* function in the *metafor* package in R [14]. This model accounts for both within-study variance and between-study heterogeneity. Forest plots are shown in **Figure S7**, displaying site-specific Hedges' *g* effect sizes with 95% confidence intervals, alongside the pooled meta-analytic effect across research sites. The heterogeneity between studies, i.e., the proportion of variability explained by between-site heterogeneity rather than chance, was estimated as *I*^2^ [15].

**Note S7 - Post-hoc analysis of diagnostic stability.**

As established in prior studies, the stability of diagnostic subgroups varies and is important for interpreting the subgroup analyses [16,17]. We therefore conducted a post-hoc analysis to estimate the diagnostic stability of EOS, AFP, and OTP in the context of the current study. For this, we pooled diagnostic data from adolescents with EOP with available follow-up assessments (n=169), drawn from the BARCELONA, MADRID, ROME, OXFORD, and YTOP cohorts. The cohort-weighted mean follow-up time was 2.93 years, with a cohort-level range of 1 to 8.5 years. We calculated the prospective positive predictive value (PPV) as:

where n_11_ denotes the number of individuals classified in a given diagnostic subgroup (EOS, AFP, or OTP) at both baseline and follow-up, and n_10_ denotes the number classified in that subgroup at baseline but not at follow-up. Accordingly, the PPV quantifies the prospective stability of each diagnostic subgroup [18]. For each estimate, we calculated 95% confidence intervals (CI) using the Wilson method and report the baseline number of individuals with follow-up data for the diagnostic subgroup, denoted n_1_.

The highest diagnostic stability was observed for EOS (n_1_=108; PPV=95.4%; CI=[89.6%, 98.2%]), which was higher than, or comparable to, stability estimates from prior studies in both adolescents and adults [16,17,19–22]. The estimated PPV was lower in AFP (n_1_=38; PPV=63.2%; CI=[47.3%, 76.6%]) than reported in prior studies, notably the study by Castro-Fornieles et al., (2011) where a PPV of 71.4% was reported for AFP (n_1_=21; CI=[50.0%, 86.2%]). Nonetheless, confidence intervals overlapped with prior studies, indicating overall statistical compatibility [16,17,20,22]. The estimated PPV for OTP was higher (n_1_=23; PPV=47.8%; CI=[29.2%, 67.0%]) than most prior studies and similar to the estimate reported by Jarbin & von Knorring (2003) (n_1_=6; PPV=66.7%; CI=[30.0%, 90.3%]), although this OTP group was small [22]. In EOS, 0.9% shifted to AFP, 0.9% to OTP and 2.8% were in remission at follow-up. In AFP, 26.3% shifted to EOS, 7.9% to OTP, and 2.6% were in remission at follow-up. Finally, in OTP, 21.7% shifted to EOS, 13.0% to AFP, and 17.4% did not meet criteria for a psychotic disorder at follow-up.

In summary, diagnostic stability in the post-hoc analysis was broadly consistent with prior studies: high for EOS, moderate-to-high for AFP, and lowest for OTP. Prior studies suggest that the first year after adolescent psychosis onset is a period in which diagnoses are more likely to change [23]. Furthermore, the high proportion of transitions from AFP to EOS in the present sample (26.3%) point to significant overlap across the affective and non-affective adolescent psychosis spectrum. Finally, given the propensity of individuals with OTP to transition to both EOS and AFP, as well as not meeting the criteria for a psychotic disorder at follow-up, results pertaining to this subgroup should be interpreted cautiously.

## 2. Supplementary Figures

**Figure S1. Overview of cortical regions in the Desikan-Killiany atlas.** There are 34 regions per hemisphere with locations determined by the sulcogyral folding pattern [10].

**Figure S2. Analysis overview.** Flowchart with a broad overview of the main analyses and their respective model specifications and statistical tests. Created with BioRender.

**Figure S3. Main analysis - Cortical thickness.** Cohen's *d* effect sizes for comparisons between patients with early-onset psychosis (EOP) and healthy controls from models adjusted for sex, age, and age^2^. Effect sizes are ranked by magnitude; significant differences after correction for multiple testing are marked with asterisks.

**Figure S4. Main analysis - Surface area.** Cohen's *d* effect sizes for comparisons between patients with early-onset psychosis (EOP) and healthy controls from models adjusted for sex, age, and age^2^. Effect sizes are ranked by magnitude; significant differences after correction for multiple testing are marked with asterisks.

**Figure S5. Main analysis - Cortical volume.** Cohen's *d* effect sizes for comparisons between patients with early-onset psychosis (EOP) and healthy controls from models adjusted for sex, age, and age^2^. Effect sizes are ranked by magnitude; significant differences after correction for multiple testing are marked with asterisks.

**Figure S6. Main analysis - Local Gyrification Index (LGI).** Cohen's *d* effect sizes for comparisons between patients with early-onset psychosis (EOP) and healthy controls from models adjusted for sex, age, and age^2^. Effect sizes are ranked by magnitude; significant differences after correction for multiple testing are marked with asterisks.

**Figure S7. Cortical asymmetry analyses.** The vertical bars depict Cohen's *d* effect sizes and their 95% confidence intervals for case-control comparisons in early-onset psychosis (EOP) vs healthy controls for the asymmetry index, i.e., left - right hemisphere divided by the mean, for each cortical region. Asterisks depict differences between EOP and healthy controls that were statistically significant after correction for multiple testing.

**Figure S8.** **Meta-analysis - Global cortical metrics.** Site-wise differences between early-onset psychosis (EOP) and healthy controls in global thickness, area, volume, and the Local Gyrification Index (LGI) for each hemisphere. Models were adjusted for age, age^2^, and sex. Hedges' *g* effect sizes and 95% confidence intervals are shown for each site. The diamond indicates the pooled effect size and its confidence interval across sites.

**Figure S9. Regional specificity - Cortical thickness.** Cohen's *d* effect sizes for comparisons between early-onset psychosis (EOP) and healthy controls adjusted for sex, age, and age^2^ and global cortical thickness. Effect sizes are ranked by magnitude; significant differences after correction for multiple testing are marked with asterisks.

**Figure S10. Regional specificity - Surface area.** Cohen's *d* effect sizes for comparisons between early-onset psychosis (EOP) and healthy controls adjusted for sex, age, and age^2^ and global surface area. Effect sizes are ranked by magnitude; significant differences after correction for multiple testing are marked with asterisks.

**Figure S11. Regional specificity - Cortical volume.** Cohen's *d* effect sizes for comparisons between early-onset psychosis (EOP) and healthy controls adjusted for sex, age, and age^2^ and global cortical volume. Effect sizes are ranked by magnitude; none of the regions differed significantly after correction for multiple testing.

**Figure S12. Regional specificity - Local Gyrification Index (LGI).** Cohen's *d* effect sizes for comparisons between early-onset psychosis (EOP) and healthy controls adjusted for sex, age, and age^2^ and global LGI. Effect sizes are ranked by magnitude; none of the regions differed significantly after correction for multiple testing.

**Figure S13. Case-control differences adjusted for intracranial volume (ICV).** Cohen's *d* effect sizes for regions with significant differences in cortical thickness, surface area, cortical volume, and Local Gyrification Index (LGI) between early-onset psychosis (EOP) and healthy controls in models adjusted for sex, age, age^2^, and ICV.

**Figure S14. Chlorpromazine-equivalent dose correlations.** Uncorrected partial correlations between chlorpromazine-equivalent antipsychotic medication dose and each cortical metric. Analyses were conducted for patients who were currently taking antipsychotic medication. No associations survived correction for multiple testing.

**Figure S15. PANSS positive symptom scores.** Uncorrected partial correlations between PANSS positive symptom scores and each cortical metric. No associations survived correction for multiple testing.

**Figure S16. PANSS negative symptom scores.** Uncorrected partial correlations between PANSS negative symptom scores and each cortical metric. Only the association with volume of the left lingual region was statistically significant (partial *r*=-0.21; *p_FDR_*=0.014).

**Figure S17. Sex-stratified analyses - Cortical thickness and surface area.** Cohen's *d* effect sizes for regions with significant differences from comparisons between early-onset psychosis (EOP) and healthy controls using separate models for males and females.

**Figure S18. Sex-stratified analyses - Cortical volume and Local Gyrification Index (LGI).** Cohen's *d* effect sizes for regions with significant differences from comparisons between early-onset psychosis (EOP) and healthy controls using separate models for males and females.

**Figure S19. Sex-stratified analyses - Cortical thickness.** Cohen's *d* effect sizes in the comparison between patients with early-onset psychosis (EOP) and healthy controls stratified by sex. Red bars indicate effect sizes for females and blue bars indicate effect sizes for males. Effect sizes are ordered by magnitude of effects for females.

**Figure S20. Sex-stratified analyses - Surface area.** Cohen's *d* effect sizes in the comparison between patients with early-onset psychosis (EOP) and healthy controls stratified by sex. Red bars indicate effect sizes for females and blue bars indicate effect sizes for males. Effect sizes are ordered by magnitude of effects for females.

**Figure S21. Sex-stratified analyses - Cortical volume.** Cohen's *d* effect sizes in the comparison between patients with early-onset psychosis (EOP) and healthy controls stratified by sex. Red bars indicate effect sizes for females and blue bars indicate effect sizes for males. Effect sizes are ordered by magnitude of effects for females.

**Figure S22. Sex-stratified analyses - Local Gyrification Index (LGI).** Cohen's *d* effect sizes in the comparison between patients with early-onset psychosis (EOP) and healthy controls stratified by sex. Red bars indicate effect sizes for females and blue bars indicate effect sizes for males. Effect sizes are ordered by magnitude of effects for females.

**Figure S23. Effect size comparison to adults with SCZ.** Cohen's *d e*ffect sizes from the case-control analyses for early-onset psychosis (EOP; *blue*) compared to those of the previous study on adults with schizophrenia (SCZ; *orange*). To harmonize covariates across studies, the regression models were only adjusted for age and sex. Significant differences in cortical thickness and surface area are marked with asterisks.

**Figure S24. Effect size comparison to adults with BD.** Cohen's *d e*ffect sizes from the case-control analyses for early-onset psychosis (EOP; *blue*) compared to those of the previous study on adults with bipolar disorders (BD; *purple*). To harmonize covariates across studies, the regression models for cortical thickness were only adjusted for age and sex, while surface area was also adjusted for intracranial volume (ICV). Significant differences in cortical thickness and surface area are marked with asterisks.

**Figure S25. Effect size comparison to youths with BD.** Cohen's *d e*ffect sizes from the case-control analyses for early-onset psychosis (EOP; *blue*) compared to those of the previous study on youths with bipolar disorders (BD; *green*). To harmonize covariates across studies, the regression models for cortical thickness were only adjusted for age and sex, while surface area was also adjusted for intracranial volume (ICV). Significant differences in cortical thickness and surface area are marked with asterisks.

**3. Supplementary Tables**

**Table S1. Overview of participating research sites.**

| **Cohort** | **Principal investigator** | **Institution** | **City** | **Country** | **Data inclusion period** |
| --- | --- | --- | --- | --- | --- |
| BARCELONA | Josefina Castro-Fornieles & Gisela Sugranyes | University of Barcelona | Barcelona | Spain | 2003 - 2020 |
| MADRID | Celso Arango | Hospital General Universitario Gregorio Marañón | Madrid | Spain | 2006 - 2014 |
| ROME | Fabrizio Piras | IRCCS Santa Lucia Foundation | Rome | Italy | 2012 - present |
| OXFORD | Anthony James | University of Oxford | Oxford | UK | 2005 - 2009 |
| KCL-1 | Sophia Frangou | King's College London | London | UK | 2002 - 2005 |
| KCL-2 | Anne-Kathrin J. Fett | King's College London | London | UK | 2010 - 2012 |
| UCLA | Carrie E. Bearden | University of California | Los Angeles | USA | 2011 - 2015 |
| SCAPS | Ingrid Agartz & Mathias Lundberg | Karolinska Institutet | Stockholm | Sweden | 2012 - 2019 |
| UIO-PSI | Bjørn Rishovd Rund &  Lars T. Westlye | University of Oslo | Oslo | Norway | 2005 - 2007 |
| YTOP | Ingrid Agartz, Anne M. Myhre, and Nils Eiel Steen | University Hospital of Oslo | Oslo | Norway | 2012 - present |

**Table S2. Recruitment procedures and criteria for each site.**

| **Cohort** | **Group** | **Clinical assessment** | **Recruitment information** | **Inclusion criteria** | **Exclusion criteria** |
| --- | --- | --- | --- | --- | --- |
| BARCELONA | EOP | K-SADS;  DSM-IV-TR | Referral from inpatient or outpatient units of the Department of Child and Adolescent Psychiatry and Psychology of the Hospital Clinic Barcelona | Age between 7-18 years; onset of first psychotic positive symptom within a psychotic episode before age of 18; diagnosis of a psychotic disorder per DSM-IV-TR criteria; written informed consent | Intellectual disability per DSM-IV-TR criteria (IQ < 70 & impaired functioning); pervasive developmental disorder; past history of head trauma with loss of consciousness; pregnancy |
|  | CTR | K-SADS;  DSM-IV-TR | Local catchment area via advertisements | Age between 7-18 years; written informed consent | Past history of psychotic illness; current diagnosis of any Axis-I DSM-IV-TR disorder; intellectual disability per DSM-IV-TR criteria (IQ < 70 & impaired functioning); past history of head trauma with loss of consciousness; pregnancy |
| MADRID | EOP | K-SADS;  DSM-IV-TR | Referral from adolescent inpatient unit (Hospital General Universitario Gregorio Marañón) or local clinical services (PIENSA program) | Age between 7-18 years; onset of first psychotic positive symptom within a psychotic episode before age of 18; diagnosis of a psychotic disorder per DSM-IV-TR criteria; written informed consent | Intellectual disability per DSM-IV-TR criteria (IQ < 70 & impaired functioning); pervasive developmental disorder; past history of head trauma with loss of consciousness; pregnancy |
|  | CTR | K-SADS;  DSM-IV-TR | Local catchment area via advertisements | Age between 7-18 years; written informed consent | Past history of psychotic illness; current diagnosis of any Axis-I DSM-IV-TR disorder; intellectual disability per DSM-IV-TR criteria (IQ < 70 & impaired functioning); past history of head trauma with loss of consciousness; pregnancy |
| ROME | EOP | DSM-V psychiatric and personality disorders using the SCID-5-RV and SCID-5-PD | Local catchment area and referral from adolescent psychiatric units | Age between 10-18 years; onset of first psychotic positive symptom within a psychotic episode before age of 18; suitability for MRI scanning; written informed consent | A history of alcohol or drug abuse in the two years before the assessment; lifetime drug dependence; traumatic head injury with loss of consciousness; past or present major medical illness or neurological disorders; intellectual disability; pervasive developmental disorder |
|  | CTR | DSM-V psychiatric and personality disorders using the SCID-5-RV and SCID-5-PD | Local catchment area via advertisements | Matched to patients; age between 10-18 years; suitability for MRI scanning; written informed consent | A history of alcohol or drug abuse in the two years before the assessment; lifetime drug dependence; traumatic head injury with loss of consciousness; past or present major medical illness or neurological disorders; any psychiatric disorder or intellectual disability |
| OXFORD | EOP | K-SADS-PL;  DSM-IV | Local adolescent psychiatric units | Psychotic disorders included: schizophrenia | Moderate mental impairment; a history of substance abuse or pervasive developmental disorder; significant head injury; neurological disorder or major medical disorder |
|  | CTR | KSADS-PL | Local general practitioners’ practice | Healthy adolescents | Any medical/emotional/behavioral disorders; moderate mental impairment; a history of substance abuse or pervasive developmental disorder; significant head injury; neurological disorder or major medical disorder |
| KCL-1 | EOP | Structured Clinical Interview for DSM-IV (SCID) Axis I Disorders | Local clinical services | DSM IV Schizophrenia, 12-19 years old, onset of schizophrenia before age 18, no comorbid Axis I diagnosis, no mental retardation | History of head injury, current substance misuse, any medical condition, history of hereditary disease of the nervous system |
|  | CTR | Structured Clinical Interview for DSM-IV (SCID) Axis I Disorders | Local area via advertisements | Age 12-19, no personal history of psychiatric disorders, no family history of psychosis in first-degree relatives | History of head injury, current substance misuse, any medical condition, history of hereditary disease of the nervous system |
| KCL-2 | EOP | ICD-10 diagnosed by treating clinician | Patients were recruited via consultant psychiatrists and via the Mental Health Research Network (MHRN) in South London and Maudsley, North East London, and South Essex Partnership University NHS Foundation Trusts | Age between 13-19 years; experienced a psychotic episode according to ICD-10 criteria, as diagnosed by their clinician; good command of the English language; being able and willing to give written informed consent | Diagnosed substance use/abuse or neurological conditions. |
| KCL-2 | CTR | No diagnosis confirmed in telephone screening | Control participants were recruited from local schools, the Institute of Psychiatry volunteer database 'Mindsearch', via colleagues and previous participants | Age between 13-19 years; good command of the English language; being able and willing to give written informed consent; no personal or family history of a psychotic illness | Diagnosed substance use/abuse or neurological conditions or a history of a psychiatric diagnosis and a family history of psychosis. |
| UCLA | EOP | DSM-IV. SCID Axis I Disorders | In-and outpatient clinics for child and adolescent mental health in the Greater Los Angeles area/website/local advertisements | Psychotic disorders included: schizophrenia spectrum disorder; age between 12-18 years; informed consent; no MRI contra-indications | Substance-induced psychotic disorder; IQ < 70; previous moderate to severe head injury; significant comorbid medial/neurological condition and/or history of head trauma with loss of consciousness |
|  | CTR | DSM-IV. SCID Axis I Disorders | Local advertisements (online/brochures) in the Los Angeles areas | Matched to patients; age between 12-18 years; no history of major mental disorders; informed consent; no MRI contra-indications | History of mental health issues (contact with specialist services); first-degree relatives with a history of psychotic disorders; IQ < 70; significant comorbid medial/neurological condition and/or history of head trauma with loss of consciousness |
| SCAPS | EOP | DSM-IV | Specialist care unit of psychosis and bipolar disorder in the department of Child and Adolescent Psychiatry in Stockholm. Sweden | Psychotic disorders included: schizophrenia. schizoaffective disorder. psychotic depression; unspecified psychosis; bipolar I and II disorder; age between 12-18 years | Substance-induced psychotic disorder; IQ < 70; previous moderate to severe head injury; organic brain disease |
|  | CTR |  | Invitation by letter after random draw from the Swedish National Registry | Age between 12-18 years. good command of the Swedish language to complete interview and neurocognitive tests | History of mental health issues (contact with specialist services); previous or current use of psychotropic medication; first-degree relatives with a history of psychotic disorders; IQ < 70; previous moderate to severe head injury; organic brain disease |
| UIO-PSI | EOP | SCID-I, mod A-D; PANSS; GAF Split version | Recruited by clinicians at in- and outpatient child- and adolescent mental health clinics in Southern Norway | Age 12-18; broad schizophrenia spectrum disorder | Psychosis not otherwise specified (NOS); history of head injury; IQ < 70 |
|  | CTR | Screened with MINI, screening mod | Recruited from schools in the patient catchment area, some from the database of the Norwegian Central Bureau of Statistics |  |  |
| YTOP | EOP | K-SADS-PL (2009)/DSM-IV | In-and outpatient clinics for child and adolescent mental health in the greater Oslo area | Age between 12-18 years; diagnosis of psychotic disorder; good command of the Norwegian language to complete interview and neurocognitive tests | Substance-induced psychotic disorder; IQ < 70; previous moderate to severe head injury; organic brain disease |
|  | CTR | K-SADS-PL (2009) | Invitation by letter after random draw from the Norwegian National Registry | Age between 12-18 years; good command of the Norwegian language to complete interview and neurocognitive tests | History of mental health issues (contact with specialist services); previous or current use of psychotropic medication; first-degree relatives with a history of psychotic disorders; IQ < 70; previous moderate to severe head injury; organic brain disease |

**Abbreviations:** EOP = early-onset psychosis; CTR = healthy controls; DSM = Structured Clinical Interview for Diagnostic and Statistical Manual of Mental Disorder; SCID = Structured Clinical Interview for DSM Disorders; K-SADS = Kiddie Schedule for Affective Disorders and Schizophrenia (PL = present and lifetime version); PANSS = Positive and Negative Syndrome Scale; IQ = intelligence quotient; MRI = magnetic resonance imaging.

**Table S3. Demographic and clinical information stratified by site.**

| **Cohort** | **Group** | **N** | **Age** | **Sex (Female)** | **Handedness (R/L/M)** | **IQ** | **FullDx (EOS/AFP/OTP)** | **PANSS Positive** | **PANSS Negative** | **AAO** | **DOI** | **CPZ** | **AP** | **LIT** | **AD** | **AE** |
| --- | --- | --- | --- | --- | --- | --- | --- | --- | --- | --- | --- | --- | --- | --- | --- | --- |
| BARCELONA | CTR | 103 | 16.3 [14.7, 17.4] | 58  (56.3%) | 25/6/0 | 104.0 [94.0, 110.8] | N.A. | N.A. | N.A. | N.A. | N.A. | N.A. | N.A. | N.A. | N.A. | N.A. |
| BARCELONA | EOP | 166 | 16.4 [15.1, 17.3] | 81  (48.8%) | 52/5/1 | 84.0 [76.0, 96.0] | 76/72/18 | 20 [16.0, 25.0] | 15 [10.5, 21.0] | 15.8 [14.6, 17.0] | 0.2 [0.1, 0.4] | 200.0 [150.0, 350.0] | 158 (95.2%) | 25 (15.1%) | 49 (29.5%) | 4 (2.4%) |
| MADRID | CTR | 25 | 16.0 [13.0, 17.0] | 12  (48.0%) | 20/2/0 | 110.0 [103.0, 119.0] | N.A. | N.A. | N.A. | N.A. | N.A. | N.A. | N.A. | N.A. | N.A. | N.A. |
| MADRID | EOP | 28 | 16.5 [14.0, 17.0] | 6  (21.4%) | 23/2/0 | 65.0 [54.0, 95.0] | 24/4/0 | 23.5 [19.8, 30.5] | 23 [16.0, 29.0] | 16.0 [14.8, 17.0] | 0.3 [0.2, 0.4] | NA | 28 (100.0%) | 0 | 2 (7.1%) | 1 (3.6%) |
| ROME | CTR | 12 | 13.0 [12.8, 14.2] | 7  (58.3%) | 10/2/0 | N.A. | N.A. | N.A. | N.A. | N.A. | N.A. | N.A. | N.A. | N.A. | N.A. | N.A. |
| ROME | EOP | 20 | 16.0 [15.0, 18.0] | 7  (35.0%) | 17/3/0 | N.A. | 16/0/4 | 11.5 [4.2, 20.8] | 27.5 [11.5, 42.5] | 15.0 [14.0, 17.0] | 1.0 [1.0, 1.2] | 200.0 [133.3, 283.3] | 18 (90.0%) | 0 | 3 (15.0%) | 3 (15.0%) |
| OXFORD | CTR | 33 | 16.3 [15.2, 17.2] | 18  (54.5%) | 29/4/0 | 109.0 [102.0, 121.0] | N.A. | N.A. | N.A. | N.A. | N.A. | N.A. | N.A. | N.A. | N.A. | N.A. |
| OXFORD | EOP | 36 | 16.6 [15.5, 17.0] | 17  (47.2%) | 28/6/1 | 85.5 [81.0, 100.2] | 36/0/0 | 23 [20.8, 24.2] | 16 [14.0, 18.0] | 14.7 [13.4, 15.7] | 1.4 [0.9, 2.4] | 300.0 [200.0, 412.0] | 36 (100.0%) | 0 | N.A. | 3 (8.3%) |
| KCL-1 | CTR | 18 | 16.0 [15.2, 17.2] | 7  (38.9%) | 16/2/0 | 100.5 [96.2, 104.5] | N.A. | N.A. | N.A. | N.A. | N.A. | N.A. | N.A. | N.A. | N.A. | N.A. |
| KCL-1 | EOP | 15 | 16.6 [15.9, 17.4] | 5  (33.3%) | 15/0/0 | 98.0 [93.5, 104.0] | 15/0/0 | 10 [7.5, 13.5] | 14 [12.0, 15.5] | 15.0 [14.1, 15.8] | 1.6 [1.0, 3.0] | N.A. | N.A. | N.A. | N.A. | N.A. |
| UCLA | CTR | 23 | 15.0 [13.0, 17.0] | 9  (39.1%) | 18/1/3 | 117.0 [107.0, 127.0] | N.A. | N.A. | N.A. | N.A. | N.A. | N.A. | N.A. | N.A. | N.A. | N.A. |
| UCLA | EOP | 28 | 16.0 [13.8, 17.0] | 15  (53.6%) | 25/1/2 | 105.0 [90.8, 113.5] | 17/7/4 | 22.5 [12.8, 32.8] | 26 [20.2, 39.8] | 13.0 [12.2, 14.8] | 1.0 [0.2, 2.8] | 200.0 [50.0, 325.0] | 16 (57.1%) | 1 (3.6%) | 14 (50.0%) | 2 (7.1%) |
| SCAPS | CTR | 24 | 16.9 [16.2, 17.7] | 19  (79.2%) | 22/2/0 | N.A. | N.A. | N.A. | N.A. | N.A. | N.A. | N.A. | N.A. | N.A. | N.A. | N.A. |
| SCAPS | EOP | 32 | 16.7  [15.6, 17.6] | 18  (56.2%) | 7/1/0 | N.A. | 2/19/11 | N.A. | N.A. | 15.2 [14.1, 16.6] | 1.0 [0.1, 2.4] | 166.5 [16.8, 258.5] | N.A. | N.A. | N.A. | N.A. |
| UIO-PSI | CTR | 31 | 15.0  [14.5, 17.0] | 16  (51.6%) | 28/3/0 | 108.0 [97.5, 116.5] | N.A. | N.A. | N.A. | N.A. | N.A. | N.A. | N.A. | N.A. | N.A. | N.A. |
| UIO-PSI | EOP | 21 | 16.0  [15.3, 17.0] | 12  (57.1%) | 19/2/0 | 98.0 [81.0, 109.0] | 14/0/7 | 13 [10.0, 16.5] | 13 [9.5, 15.0] | 15.0 [14.0, 16.0] | 1.2 [0.8, 2.2] | 67.0 [0.0, 300.0] | 10 (47.6%) | NA | 3 (14.3%) | 0 |
| YTOP | CTR | 69 | 16.2  [15.3, 17.4] | 38  (55.1%) | 65/4/0 | 107.0 [96.5, 114.0] | N.A. | N.A. | N.A. | N.A. | N.A. | N.A. | N.A. | N.A. | N.A. | N.A. |
| YTOP | EOP | 41 | 16.5  [15.7, 17.3] | 30  (73.2%) | 35/3/0 | 100.5 [88.0, 108.0] | 23/2/16 | 16 [14.0, 20.0] | 21 [14.0, 23.0] | 15.2 [13.0, 16.0] | 1.1 [0.7, 2.2] | 75.0 [0.0, 133.8] | 19 (46.3%) | 0 | 3 (7.3%) | 0 |

**Abbreviations:** EOP = early-onset psychosis; CTR = healthy controls; EOS = early-onset schizophrenia; AFP = affective psychosis; OTP = other psychotic disorders; PANSS = Positive and Negative Syndrome Scale; AAO = age at onset; DOI = duration of illness; AP = antipsychotic medication; CPZ = chlorpromazine-equivalent antipsychotic medication dose; LIT = lithium; AD = antidepressant medication; AE = antiepileptic medication; N.A. = not applicable. Handedness is given as number of right-handed, left-handed, and mixed individuals, among individuals where handedness information was available.

***Notes:*** The demographic and clinical information is given for the final mega-analysis dataset after 42 participants were excluded after quality control. The additional dataset used in the meta-analysis is described in **Table S5**.

**Table S4. Definitions of age at onset and duration of illness for each site.**

| **Cohort** | **Age at onset (AAO)** | **Duration of illness (DOI)** |
| --- | --- | --- |
| BARCELONA | Presence of positive psychotic symptoms within a psychotic episode | DOI = Age at MRI - AAO |
| MADRID | Presence of positive psychotic symptoms within a psychotic episode | DOI = Age at MRI - AAO |
| ROME | Age at onset of first positive psychotic symptoms, diagnosed by mental health or medical professional | DOI = Age at MRI - AAO |
| OXFORD | Age at onset of first positive psychotic symptoms, determined during the diagnostic Kiddie-SADS interview; supplemented by medical record review if needed. | DOI = Age at MRI - AAO |
| KCL-1 | Age at onset of first positive psychotic symptoms, determined during the diagnostic Kiddie-SADS interview; supplemented by medical record review if needed. | DOI = Age at MRI - AAO |
| KCL-2 | Age at first diagnosis, confirmed with treating clinician. | DOI = Age at MRI - AAO |
| UCLA | Age of first overt psychotic symptoms, determined via SIPS/SCID interview; supplemented by collateral informant if needed. | DOI = Age at MRI - AAO |
| SCAPS | Age at onset of first psychotic episode, determined by medical record review. | DOI = Age at MRI - AAO |
| UIO-PSI | Age at onset of first positive psychotic symptoms, determined during the diagnostic interview. | DOI = Age at MRI - AAO |
| YTOP | Age at onset of first positive psychotic symptoms, determined during the diagnostic Kiddie-SADS interview; supplemented by medical record review if needed. | DOI = Age at MRI - AAO |

**Notes:** The KCL-2 dataset was only used in meta-analyses on main group differences and not analyses on associations with age at onset or duration of illness, but the operational definitions are reported for completeness.

**Table S5.** **Demographic and clinical information for KCL-2 dataset.**

|  | **CTR (n=25)** | **EOP (n=20)** |
| --- | --- | --- |
| **Age [years]** | 16.36 (1.6) | 16.95 (1.2) |
| **Sex [female]** | 0 (0%) | 0 (0%) |
| **Parental education [years]** | N.A. | N.A. |
| **Handedness [right]** | 25 (100.0%) | 20 (100.0%) |
| **IQ** | N.A. | N.A. |
| **Age at onset [years]** | N.A. | 16.3 (1.4) |
| **Duration of illness [years]** | N.A. | 1.1 (0.8) |
| **PANSS Positive** | N.A. | N.A. |
| **PANSS Negative** | N.A. | N.A. |
| **SAPS** | N.A. | N.A. |
| **SANS** | N.A. | N.A. |
| **AP use [yes]** | N.A. | 17 (85%) |
| **CPZ [mg/day]** | N.A. | N.A. |
| **Antiepileptic use [yes]** | N.A. | 3 (15%) |
| **Antidepressant use [yes]** | N.A. | 5 (25%) |
| **Lithium use [yes]** | N.A. | 0 (0%) |

**Abbreviations:** PANSS = Positive and Negative Syndrome Scale, AP = antipsychotic medication, CPZ = chlorpromazine-equivalent antipsychotic medication dose, N.A. = not applicable.

***Notes:*** Continuous variables are given as means (standard deviations) and categorical variables as counts (percentage of non-missing). This dataset was used only in the meta-analyses and information is reported for healthy controls (CTR) and the early-onset psychosis (EOP).

**Table S6. MRI acquisition parameters for each site.**

| **Cohort** | **Scanner** | **Field strength** | **Scanner model** | **Sequence**  **description** | **Acquisition direction** | **Resolution (mm^3^)** | **TI (ms)** | **TE (ms)** | **TR (ms)** | **Flip**  **angle (°)** |
| --- | --- | --- | --- | --- | --- | --- | --- | --- | --- | --- |
| BARCELONA | 1 | 1.5T | GE Genesis Signa | 3D T1-weighted | Axial | 0.97x0.97x1.5 | 300 | 5.168 | 12 | 20 |
|  | 2 | 3T | Siemens TrioTim | MPRAGE sequence | Sagittal | 1x1x0.95 | 900 | 3.01 | 2300 | 9 |
| MADRID | 4 | 1.5T | Philips | 3D T1-weighted | Sagittal | 1x0.94x0.94 | - | 9.2 | 25 | - |
| ROME | 5 | 3T | Philips Achieva | MPRAGE sequence | Sagittal | 0.5x0.5x0.9 | - | 5.3 | 11 | 8 |
| OXFORD | 6 | 1.5T | Siemens | 3D T1-weighted | Coronal | 1x1x1 | - | 5.6 | 12 | 19 |
| KCL-1 | 7 | 1.5T | GE | 3D T1-weighted | Coronal | 0.86x0.86x1.5 | 450 | 3.0 | 14 | 20 |
| KCL-2 | 8 | 3T | GE Signa | 3D T1-weighted | Coronal | 1.1x1.1x1.1 | - | 2.8 | 7 | 20 |
| UCLA | 9 | 3T | Siemens Tim Trio | 3D T1-weighted | Sagittal | 1x1x1 | 900 | 2.91 | 2300 | 9 |
| SCAPS | 10 | 3T | GE Discovery MR750 | BRAVO sequence | Sagittal | 1x1x1.2 | - | 3.06 | 7.9 | 12 |
| UIO-PSI | 11 | 1.5T | Siemens Sonata | 3D-SPGR sequence | Coronal | 1x1x1 | 1000 | 3.93 | 2730 | 7 |
| YTOP | 12 | 3T | GE Signa HDxt | FSPGR sequence | Sagittal | 1x1x1.2 | 450 | MinFull | 7.8 | 12 |
|  | 13 | 3T | GE Discovery MR750 | BRAVO sequence | Sagittal | 1x1x1 | 450 | 3.18 | 8.16 | 12 |

**Abbreviations:** TI = inversion time; TE = echo time; TR = repetition time.

**Table S7. Model summaries for global cortical metrics.**

|  | **Left hemisphere** | | | | | | | |
| --- | --- | --- | --- | --- | --- | --- | --- | --- |
|  | **Global Cortical Thickness** | | **Global Surface Area** | | **Global Cortical Volume** | | **Global LGI** | |
| ***Predictors*** | ***Estimates*** | ***p*** | ***Estimates*** | ***p*** | ***Estimates*** | ***p*** | ***Estimates*** | ***p*** |
| **Intercept** | 3.18  (-0.80 – 7.15) | 0.117 | -4.69  (-8.32 – -1.06) | 0.011 | -3.41  (-6.90 – 0.09) | 0.056 | 1.08  (-2.69 – 4.85) | 0.575 |
| **Age (years)** | -0.15  (-0.68 – 0.37) | 0.574 | 0.72  (0.24 – 1.20) | 0.003 | 0.67  (0.21 – 1.13) | 0.004 | 0.12  (-0.37 – 0.62) | 0.624 |
| **Age^2^** | -0.00  (-0.02 – 0.02) | 0.81 | -0.02  (-0.04 – -0.01) | 0.004 | -0.03  (-0.04 – -0.01) | 0.001 | -0.01  (-0.03 – 0.01) | 0.245 |
| **Sex (female)** | -0.11  (-0.24 – 0.03) | 0.121 | -1.09  (-1.21 – -0.96) | <0.001 | -1.07  (-1.19 – -0.96) | <0.001 | -0.77  (-0.89 – -0.64) | <0.001 |
| **EOP** | -0.33  (-0.46 – -0.20) | <0.001 | -0.35  (-0.47 – -0.23) | <0.001 | -0.47  (-0.58 – -0.35) | <0.001 | -0.34  (-0.46 – -0.21) | <0.001 |
| **R^2^ / R^2^ adjusted** | 0.176 / 0.171 | | 0.314 / 0.310 | | 0.363 / 0.359 | | 0.259 / 0.255 | |
|  | **Right hemisphere** | | | | | | | |
|  | **Global Cortical Thickness** | | **Global Surface Area** | | **Global Cortical Volume** | | **Global LGI** | |
| ***Predictors*** | ***Estimates*** | ***p*** | ***Estimates*** | ***p*** | ***Estimates*** | ***p*** | ***Estimates*** | ***p*** |
| **Intercept** | 3.31  (-0.64 – 7.27) | 0.1 | -4.34  (-7.96 – -0.72) | 0.019 | -3.14  (-6.65 – 0.37) | 0.08 | -0.61  (-4.31 – 3.10) | 0.748 |
| **Age (years)** | -0.16  (-0.68 – 0.36) | 0.552 | 0.67  (0.19 – 1.14) | 0.006 | 0.63  (0.17 – 1.10) | 0.008 | 0.34  (-0.14 – 0.83) | 0.168 |
| **Age^2^** | -0.00  (-0.02 – 0.01) | 0.791 | -0.02  (-0.04 – -0.01) | 0.007 | -0.02  (-0.04 – -0.01) | 0.002 | -0.02  (-0.03 – -0.00) | 0.044 |
| **Sex (female)** | -0.08  (-0.21 – 0.06) | 0.258 | -1.10  (-1.22 – -0.97) | <0.001 | -1.07  (-1.19 – -0.96) | <0.001 | -0.81  (-0.94 – -0.69) | <0.001 |
| **EOP** | -0.28  (-0.42 – -0.15) | <0.001 | -0.34  (-0.46 – -0.22) | <0.001 | -0.45  (-0.57 – -0.33) | <0.001 | -0.44  (-0.57 – -0.32) | <0.001 |
| **R^2^ / R^2^ adjusted** | 0.185 / 0.180 | | 0.317 / 0.313 | | 0.357 / 0.354 | | 0.285 / 0.281 | |

***Notes:*** Summaries from the main model adjusted for sex, age, age^2^ with diagnostic status (CTR/EOP) as the variable of interest. The outcome variables are mean-centered and scaled to facilitate comparison across cortical metrics.

**Table S8. Model fit for covariates and diagnostic status for global cortical metrics.**

|  | **Left hemisphere** | | | |
| --- | --- | --- | --- | --- |
|  | **Total Cortical Thickness** | **Total Surface Area** | **Total Cortical Volume** | **Total LGI** |
| **Base model R^2^  (Age, Age^2^, Sex, Site)** | 0.150 | 0.285 | 0.311 | 0.233 |
| **Full model R^2^  (base model + Dx)** | 0.176 | 0.314 | 0.363 | 0.259 |
| **ΔR^2^ (base vs full)** | 0.026 | 0.029 | 0.052 | 0.027 |
| ***F* (ΔR^2^)** | 22.2 | 30.1 | 57.6 | 25.7 |
| ***p*-value (ΔR^2^)** | 2.95E-06 | 5.82E-08 | 9.93E-14 | 5.15E-07 |
|  | **Right hemisphere** | | | |
|  | **Total Cortical Thickness** | **Total Surface Area** | **Total Cortical Volume** | **Total LGI** |
| **Base model R^2^  (Age, Age^2^, Sex, Site)** | 0.166 | 0.290 | 0.309 | 0.239 |
| **Full model R^2^  (base model + Dx)** | 0.185 | 0.318 | 0.357 | 0.286 |
| **ΔR^2^ (base vs full)** | 0.019 | 0.027 | 0.048 | 0.046 |
| ***F* (ΔR^2^)** | 16.6 | 28.6 | 53.7 | 46.1 |
| ***p*-value (ΔR^2^)** | 5.18E-05 | 1.20E-07 | 6.40E-13 | 2.41E-11 |

***Notes:*** Model fit for a base regression model adjusted for sex, age, age^2^, and site, compared to a full regression model that also includes diagnostic status (Dx; CTR/EOP). Rows report unadjusted R^2^ for the base and full models, where R^2^ change is the variance uniquely explained by Dx over covariates, *F* (ΔR^2^) is the omnibus *F*-statistic comparing the full vs base model, and the *p*-value is the significance of that comparison. Site effects were negligible due to ComBat harmonization.

**Table S9. Correlations between case-control alterations in diagnostic subgroups.**

|  | **Cortical thickness** | | |
| --- | --- | --- | --- |
|  | **EOS** | **AFP** | **OTP** |
| **EOS** | 1 | 0.78 | 0.53 |
| **AFP** | 0.78 | 1 | 0.59 |
| **OTP** | 0.53 | 0.59 | 1 |
|  | **Surface area** | | |
|  | **EOS** | **AFP** | **OTP** |
| **EOS** | 1 | 0.57 | 0.54 |
| **AFP** | 0.57 | 1 | 0.33 |
| **OTP** | 0.54 | 0.33 | 1 |
|  | **Cortical volume** | | |
|  | **EOS** | **AFP** | **OTP** |
| **EOS** | 1 | 0.70 | 0.69 |
| **AFP** | 0.70 | 1 | 0.45 |
| **OTP** | 0.69 | 0.45 | 1 |
|  | **Local Gyrification Index** | | |
|  | **EOS** | **AFP** | **OTP** |
| **EOS** | 1 | 0.58 | 0.60 |
| **AFP** | 0.58 | 1 | 0.68 |
| **OTP** | 0.60 | 0.68 | 1 |

***Notes:*** Pearson correlation coefficients for the Cohen's *d* effect sizes in the case-control analysis for the diagnostic subgroup analyses for cortical thickness, surface area, cortical volume, and the Local Gyrification Index (LGI).

**Table S10. Model summaries for AP use differences in global cortical metrics.**

|  | **Left hemisphere** | | | | | | | |
| --- | --- | --- | --- | --- | --- | --- | --- | --- |
|  | **Global Cortical Thickness** | | **Global Surface Area** | | **Global Cortical Volume** | | **Global LGI** | |
| ***Predictors*** | ***Estimates*** | ***p*** | ***Estimates*** | ***p*** | ***Estimates*** | ***p*** | ***Estimates*** | ***p*** |
| **Intercept** | 2.82  (-3.64 – 9.27) | 0.391 | -6.93  (-12.88 – -0.98) | 0.023 | -4.89  (-10.65 – 0.88) | 0.096 | 2.31  (-3.96 – 8.58) | 0.468 |
| **Age (years)** | -0.09  (-0.93 – 0.76) | 0.837 | 0.98  (0.20 – 1.76) | 0.014 | 0.84  (0.09 – 1.59) | 0.029 | -0.08  (-0.90 – 0.74) | 0.849 |
| **Age^2^** | -0.01  (-0.03 – 0.02) | 0.709 | -0.03  (-0.06 – -0.01) | 0.015 | -0.03  (-0.06 – -0.01) | 0.014 | -0.00  (-0.03 – 0.02) | 0.873 |
| **Sex (female)** | -0.15  (-0.34 – 0.05) | 0.152 | -1.06  (-1.24 – -0.88) | <0.001 | -1.10  (-1.28 – -0.93) | <0.001 | -0.79  (-0.98 – -0.60) | <0.001 |
| **AP use (yes)** | 0.03  (-0.27 – 0.33) | 0.839 | -0.13  (-0.41 – 0.14) | 0.343 | -0.11  (-0.37 – 0.16) | 0.432 | -0.12  (-0.41 – 0.17) | 0.433 |
| **R^2^ / R^2^ adjusted** | 0.153 / 0.143 | | 0.281 / 0.272 | | 0.325 / 0.317 | | 0.201 / 0.192 | |
|  | **Right hemisphere** | | | | | | | |
|  | **Global Cortical Thickness** | | **Global Surface Area** | | **Global Cortical Volume** | | **Global LGI** | |
| ***Predictors*** | ***Estimates*** | ***p*** | ***Estimates*** | ***p*** | ***Estimates*** | ***p*** | ***Estimates*** | ***p*** |
| **Intercept** | 2.19  (-4.22 – 8.60) | 0.502 | -5.91  (-11.82 – -0.01) | 0.05 | -4.48  (-10.23 – 1.28) | 0.127 | -0.03  (-6.17 – 6.11) | 0.992 |
| **Age (years)** | 0.01  (-0.83 – 0.84) | 0.99 | 0.84  (0.07 – 1.61) | 0.033 | 0.78  (0.03 – 1.54) | 0.041 | 0.24  (-0.57 – 1.04) | 0.565 |
| **Age^2^** | -0.01  (-0.04 – 0.02) | 0.534 | -0.03  (-0.05 – -0.00) | 0.037 | -0.03  (-0.05 – -0.00) | 0.021 | -0.01  (-0.04 – 0.01) | 0.356 |
| **Sex (female)** | -0.10  (-0.30 – 0.10) | 0.327 | -1.08  (-1.26 – -0.90) | <0.001 | -1.11  (-1.29 – -0.93) | <0.001 | -0.88  (-1.07 – -0.69) | <0.001 |
| **AP use (yes)** | 0.02  (-0.28 – 0.32) | 0.903 | -0.11  (-0.38 – 0.17) | 0.443 | -0.08  (-0.35 – 0.19) | 0.55 | -0.16  (-0.45 – 0.12) | 0.262 |
| **R^2^ / R^2^ adjusted** | 0.165 / 0.155 | | 0.328 / 0.320 | | 0.165 / 0.155 | | 0.328 / 0.320 | |

***Notes:*** Summaries from models fitted for patients only, where antipsychotic medication (AP) use was available, adjusted for sex, age, age^2^ with AP use (yes/no) as variable of interest. The outcomes were mean-centered and scaled to facilitate comparison across cortical metrics.

**Table S11. Model summaries for CPZ associations with global cortical metrics.**

|  | **Left hemisphere** | | | | | | | |
| --- | --- | --- | --- | --- | --- | --- | --- | --- |
|  | **Global Cortical Thickness** | | **Global Surface Area** | | **Global Cortical Volume** | | **Global LGI** | |
| ***Predictors*** | ***Estimates*** | ***p*** | ***Estimates*** | ***p*** | ***Estimates*** | ***p*** | ***Estimates*** | ***p*** |
| **Intercept** | 1.86  (-6.14 – 9.86) | 0.647 | -7.87  (-15.03 – -0.71) | 0.031 | -5.72  (-12.77 – 1.33) | 0.111 | 4.58  (-2.99 – 12.15) | 0.235 |
| **Age (years)** | 0.01  (-1.03 – 1.05) | 0.982 | 1.08  (0.15 – 2.01) | 0.023 | 0.92  (0.00 – 1.84) | 0.049 | -0.37  (-1.35 – 0.61) | 0.459 |
| **Age^2^** | -0.01  (-0.04 – 0.03) | 0.675 | -0.03  (-0.06 – -0.00) | 0.025 | -0.03  (-0.06 – -0.00) | 0.031 | 0.01  (-0.02 – 0.04) | 0.654 |
| **Sex (female)** | -0.09  (-0.32 – 0.14) | 0.441 | -1.06  (-1.26 – -0.85) | <0.001 | -1.08  (-1.29 – -0.88) | <0.001 | -0.77  (-0.99 – -0.55) | <0.001 |
| **CPZ** | -0.05  (-0.10 – 0.01) | 0.127 | 0.00  (-0.05 – 0.05) | 0.987 | -0.02  (-0.07 – 0.03) | 0.469 | -0.04  (-0.10 – 0.01) | 0.131 |
| **R^2^ / R^2^ adjusted** | 0.111 / 0.098 | | 0.287 / 0.276 | | 0.310 / 0.299 | | 0.203 / 0.191 | |
|  | **Right hemisphere** | | | | | | | |
|  | **Global Cortical Thickness** | | **Global Surface Area** | | **Global Cortical Volume** | | **Global LGI** | |
| ***Predictors*** | ***Estimates*** | ***p*** | ***Estimates*** | ***p*** | ***Estimates*** | ***p*** | ***Estimates*** | ***p*** |
| **Intercept** | 0.60  (-7.33 – 8.53) | 0.882 | -6.89  (-13.97 – 0.20) | 0.057 | -5.51  (-12.52 – 1.50) | 0.123 | 1.38  (-6.04 – 8.80) | 0.715 |
| **Age (years)** | 0.19  (-0.84 – 1.22) | 0.714 | 0.94  (0.02 – 1.87) | 0.044 | 0.89  (-0.02 – 1.80) | 0.054 | 0.05  (-0.92 – 1.01) | 0.924 |
| **Age^2^** | -0.01  (-0.05 – 0.02) | 0.423 | -0.03  (-0.06 – -0.00) | 0.049 | -0.03  (-0.06 – -0.00) | 0.035 | -0.01  (-0.04 – 0.02) | 0.698 |
| **Sex (female)** | -0.05  (-0.28 – 0.18) | 0.683 | -1.09  (-1.29 – -0.88) | <0.001 | -1.10  (-1.30 – -0.90) | <0.001 | -0.88  (-1.09 – -0.66) | <0.001 |
| **CPZ** | -0.04  (-0.10 – 0.02) | 0.182 | -0.00  (-0.05 – 0.05) | 0.97 | -0.02  (-0.07 – 0.03) | 0.495 | -0.03  (-0.09 – 0.02) | 0.258 |
| **R^2^ / R^2^ adjusted** | 0.126 / 0.113 | | 0.302 / 0.292 | | 0.317 / 0.307 | | 0.235 / 0.223 | |

***Notes:*** Summaries from models fitted for patients only, where information on chlorpromazine (CPZ) equivalent dose was available, adjusted for sex, age, age^2^, with CPZ as the variable of interest. The outcome variables are mean-centered and scaled to facilitate comparison across cortical metrics and CPZ was rescaled by 100 (CPZ/100) so that regression coefficients represent change in the outcome per 100 mg CPZ.

**Table S12. Statistical comparisons between correlation coefficients.**

| **Correlation 1 (r_1_)** | **Correlation 2 (r_2_)** | **r_1_** | **r_2_** | **z** | **p** |  |
| --- | --- | --- | --- | --- | --- | --- |
| EOP vs SCZ (adults) - Cortical thickness | EOP vs BD (adults) - Cortical thickness | 0.62 | 0.61 | 0.24 | 0.809 |  |
| EOP vs SCZ (adults) - Cortical thickness | EOP vs BD (youths) - Cortical thickness | 0.62 | 0.35 | 2.33 | 0.0199 | * |
| EOP vs BD (adults) - Cortical thickness | EOP vs BD (youths) - Cortical thickness | 0.61 | 0.35 | 2.4 | 0.0164 | * |
| EOP vs SCZ (adults) - Cortical thickness | EOP vs SCZ (adults) - Surface area | 0.62 | 0.49 | 1.16 | 0.247 |  |
| EOP vs BD (adults) - Cortical thickness | EOP vs BD (adults) - Surface area | 0.61 | 0.12 | 3.4 | <0.001 | * |
| EOP vs BD (youths) - Cortical thickness | EOP vs BD (youths) - Surface area | 0.35 | 0.05 | 1.77 | 0.0763 |  |
| EOP vs SCZ (adults) - Surface area | EOP vs BD (adults) - Surface area | 0.49 | 0.18 | 2.38 | 0.0172 | * |
| EOP vs SCZ (adults) - Surface area | EOP vs BD (youths) - Surface area | 0.49 | 0.05 | 2.76 | 0.00578 | * |
| EOP vs BD (adults) - Surface area | EOP vs BD (youths) - Surface area | 0.12 | 0.05 | 0.41 | 0.683 |  |

***Notes:*** Statistical comparisons between Pearson correlation coefficients between early-onset psychosis (EOP) and adults with schizophrenia (SCZ), adults with bipolar disorders (BD), and youths with BD. The comparisons were conducted using Steiger's test and correlation coefficients that differed significantly are denoted with an asterisk.

**Table S13. EOP vs SCZ (adults) - Cortical thickness. Significant effect size differences.**

|  | **Cortical thickness - EOP vs SCZ (adults)** | | | | | | |
| --- | --- | --- | --- | --- | --- | --- | --- |
|  | **Left hemisphere** | | |  | **Right hemisphere** | | |
| **Region** | **EOP** | **SCZ** | **p-value** | **Region** | **EOP** | **SCZ** | **p-value** |
| cuneus | 0.14 | -0.20 | 9.36×10^-5^ | bankssts | -0.11 | -0.36 | 2.73×10^-3^ |
| frontalpole | -0.04 | -0.21 | 3.21×10^-2^ | cuneus | -0.02 | -0.23 | 1.42×10^-2^ |
| fusiform | -0.26 | -0.49 | 7.98×10^-3^ | fusiform | -0.25 | -0.54 | 7.60×10^-4^ |
| insula | -0.22 | -0.41 | 2.98×10^-2^ | inferiorparietal | -0.17 | -0.35 | 4.84×10^-2^ |
| isthmuscingulate | -0.13 | -0.31 | 4.69×10^-2^ | inferiortemporal | -0.09 | -0.44 | 4.90×10^-5^ |
| lateralorbitofrontal | -0.11 | -0.40 | 1.07×10^-3^ | lateralorbitofrontal | -0.07 | -0.36 | 1.63×10^-3^ |
| lingual | -0.16 | -0.35 | 3.45×10^-2^ | lingual | -0.15 | -0.39 | 9.33×10^-3^ |
| medialorbitofrontal | -0.03 | -0.23 | 2.57×10^-2^ | medialorbitofrontal | -0.06 | -0.24 | 2.47×10^-2^ |
| middletemporal | -0.13 | -0.44 | 7.34×10^-4^ | middletemporal | -0.11 | -0.38 | 2.59×10^-3^ |
| posteriorcingulate | -0.09 | -0.30 | 1.06×10^-2^ | parahippocampal | -0.10 | -0.29 | 2.87×10^-2^ |
| rostralanteriorcingulate | 0.00 | -0.18 | 3.54×10^-2^ | parsopercularis | -0.12 | -0.42 | 3.77×10^-4^ |
| superiortemporal | -0.24 | -0.44 | 2.14×10^-2^ | parsorbitalis | -0.14 | -0.34 | 2.33×10^-2^ |
| supramarginal | -0.20 | -0.40 | 3.16×10^-2^ | parstriangularis | -0.15 | -0.37 | 1.54×10^-2^ |
| temporalpole | -0.03 | -0.25 | 1.04×10^-2^ | pericalcarine | 0.12 | -0.09 | 2.22×10^-2^ |
|  |  |  |  | temporalpole | 0.04 | -0.24 | 1.15×10^-3^ |

***Notes:*** Regions where the Cohen's *d* effect sizes from the comparison of cortical thickness in early-onset psychosis (EOP) with healthy controls differed significantly from that of adults with schizophrenia (SCZ) from the previous large-scale study from ENIGMA-SZ.

**Table S14. EOP vs SCZ (adults) - Surface area. Significant effect size differences.**

|  | **Surface area - EOP vs SCZ (adults)** | | | | | | |
| --- | --- | --- | --- | --- | --- | --- | --- |
|  | **Left hemisphere** | | |  | **Right hemisphere** | | |
| **Region** | **EOP** | **SCZ** | **p-value** | **Region** | **EOP** | **SCZ** | **p-value** |
| fusiform | -0.48 | -0.22 | 1.03×10^-3^ | medialorbitofrontal | -0.37 | -0.18 | 3.21×10^-2^ |
| inferiortemporal | -0.38 | -0.21 | 3.29×10^-2^ | middletemporal | -0.44 | -0.21 | 7.67×10^-3^ |
| insula | -0.31 | -0.12 | 2.83×10^-2^ | paracentral | -0.29 | -0.12 | 3.39×10^-2^ |
| lateraloccipital | -0.36 | -0.17 | 2.21×10^-2^ | precuneus | -0.37 | -0.15 | 6.50×10^-3^ |
| medialorbitofrontal | -0.30 | -0.11 | 2.67×10^-2^ |  |  |  |  |
| paracentral | -0.34 | -0.09 | 1.49×10^-3^ |  |  |  |  |

***Notes:*** Regions where the Cohen's *d* effect sizes from the comparison of surface area in early-onset psychosis (EOP) with healthy controls differed significantly from that of adults with schizophrenia (SCZ) from the previous large-scale study from ENIGMA-SZ.

**Table S15. EOP vs BD (adults) - Cortical thickness. Significant effect size differences.**

|  | **Cortical thickness - EOP vs BD (adults)** | | | | | | |
| --- | --- | --- | --- | --- | --- | --- | --- |
|  | **Left hemisphere** | | |  | **Right hemisphere** | | |
| **Region** | **EOP** | **BD (adults)** | **p-value** | **Region** | **EOP** | **BD (adults)** | **p-value** |
| cuneus | 0.14 | -0.06 | 1.67×10^-2^ | insula | -0.33 | -0.17 | 4.45×10^-2^ |
| medialorbitofrontal | -0.03 | -0.20 | 4.00×10^-2^ | medialorbitofrontal | -0.06 | -0.23 | 3.13×10^-2^ |
| postcentral | -0.31 | -0.10 | 9.56×10^-3^ | postcentral | -0.26 | -0.08 | 2.38×10^-2^ |

***Notes:*** Regions where the Cohen's *d* effect sizes from the comparison of cortical thickness in early-onset psychosis (EOP) with healthy controls differed significantly from that of adults with bipolar disorders (BD) from the previous large-scale study from ENIGMA-BD.

**Table S16. EOP vs BD (adults) - Surface area. Significant effect size differences.**

|  | **Surface area - EOP vs BD (adults)** | | | | | | |
| --- | --- | --- | --- | --- | --- | --- | --- |
|  | **Left hemisphere** | | |  | **Right hemisphere** | | |
| **Region** | **EOP** | **BD (adults)** | **p-value** | **Region** | **EOP** | **BD (adults)** | **p-value** |
| frontalpole | -0.14 | 0.05 | 1.84×10^-2^ | bankssts | -0.21 | -0.04 | 3.27×10^-2^ |
| fusiform | -0.39 | -0.08 | 1.14×10^-4^ | entorhinal | -0.14 | 0.10 | 4.22×10^-3^ |
| inferiortemporal | -0.27 | -0.03 | 2.61×10^-3^ | fusiform | -0.21 | -0.02 | 1.89×10^-2^ |
| lateraloccipital | -0.25 | -0.01 | 2.44×10^-3^ | inferiortemporal | -0.26 | -0.02 | 3.61×10^-3^ |
| paracentral | -0.23 | 0.01 | 2.15×10^-3^ | medialorbitofrontal | -0.24 | -0.05 | 1.47×10^-2^ |
|  |  |  |  | middletemporal | -0.34 | -0.03 | 1.40×10^-4^ |
|  |  |  |  | paracentral | -0.18 | -0.01 | 4.10×10^-2^ |
|  |  |  |  | precuneus | -0.25 | -0.01 | 3.31×10^-3^ |
|  |  |  |  | transversetemporal | -0.20 | -0.02 | 2.37×10^-2^ |

***Notes:*** Regions where the Cohen's *d* effect sizes from the comparison of surface area in early-onset psychosis (EOP) with healthy controls differed significantly from that of adults with bipolar disorders (BD) from the previous large-scale study from ENIGMA-BD.

**Table S17. EOP vs BD (youths) - Cortical thickness. Significant effect size differences.**

|  | **Cortical thickness - EOP vs BD (youths)** | | | | | | |
| --- | --- | --- | --- | --- | --- | --- | --- |
|  | **Left hemisphere** | | |  | **Right hemisphere** | | |
| **Region** | **EOP** | **BD (youths)** | **p-value** | **Region** | **EOP** | **BD (youths)** | **p-value** |
| bankssts | -0.22 | 0.00 | 1.99×10^-2^ | insula | -0.33 | -0.06 | 4.11×10^-3^ |
| inferiorparietal | -0.29 | -0.09 | 3.28×10^-2^ | lateraloccipital | -0.22 | -0.03 | 4.03×10^-2^ |
| inferiortemporal | -0.33 | -0.06 | 3.49×10^-3^ | postcentral | -0.26 | -0.07 | 4.71×10^-2^ |
| parsorbitalis | -0.25 | 0.00 | 8.77×10^-3^ | superiorfrontal | -0.37 | -0.12 | 7.20×10^-3^ |
| postcentral | -0.31 | -0.03 | 3.69×10^-3^ | superiortemporal | -0.35 | -0.10 | 9.96×10^-3^ |
| rostralmiddlefrontal | -0.30 | -0.11 | 4.26×10^-2^ | transversetemporal | -0.25 | 0.01 | 6.80×10^-3^ |
| superiorfrontal | -0.36 | -0.09 | 4.12×10^-3^ |  |  |  |  |
| superiorparietal | -0.30 | -0.07 | 1.62×10^-2^ |  |  |  |  |

***Notes:*** Regions where the Cohen's *d* effect sizes from the comparison of cortical thickness in early-onset psychosis (EOP) with healthy controls differed significantly from that of youths (i.e., < 25 years) with bipolar disorders (BD) from the previous large-scale study from ENIGMA-BD.

**Table S18. EOP vs BD (youths) - Surface area. Significant effect size differences.**

|  | **Surface area - EOP vs BD (youths)** | | | | | | |
| --- | --- | --- | --- | --- | --- | --- | --- |
|  | **Left hemisphere** | | |  | **Right hemisphere** | | |
| **Region** | **EOP** | **BD (youths)** | **p-value** | **Region** | **EOP** | **BD (youths)** | **p-value** |
| fusiform | -0.39 | 0.00 | 3.86×10^-5^ | bankssts | -0.21 | -0.01 | 2.99×10^-2^ |
| inferiortemporal | -0.27 | 0.02 | 2.13×10^-3^ | inferiorparietal | -0.12 | 0.07 | 4.20×10^-2^ |
| lateraloccipital | -0.25 | 0.03 | 2.66×10^-3^ | inferiortemporal | -0.26 | 0.02 | 3.47×10^-3^ |
| middletemporal | -0.17 | 0.04 | 2.06×10^-2^ | medialorbitofrontal | -0.24 | 0.02 | 5.50×10^-3^ |
| paracentral | -0.23 | 0.02 | 6.52×10^-3^ | middletemporal | -0.34 | 0.00 | 3.62×10^-4^ |
| superiorfrontal | -0.20 | 0.00 | 3.22×10^-2^ | precuneus | -0.25 | 0.06 | 1.14×10^-3^ |

***Notes:*** Regions where the Cohen's *d* effect sizes from the comparison of surface area in early-onset psychosis (EOP) with healthy controls differed significantly from that of youths (i.e., < 25 years) with bipolar disorders (BD) from the previous large-scale study from ENIGMA-BD.

## 4. References

1.	Kay SR, Fiszbein A, Opler LA. The Positive and Negative Syndrome Scale (PANSS) for Schizophrenia. Schizophr Bull. 1987 Jan 1;13(2):261–76.

2.	Andreasen NC. Scale for the assessment of negative symptoms (SANS). University of Iowa, Iowa City; 1983.

3.	Andreasen NC. Scale for the assessment of positive symptoms (SAPS). University of Iowa, Iowa City; 1984.

4.	Woods SW. Chlorpromazine equivalent doses for the newer atypical antipsychotics. J Clin Psychiatry. 2003 June;64(6):663–7.

5.	Leys C, Ley C, Klein O, Bernard P, Licata L. Detecting outliers: Do not use standard deviation around the mean, use absolute deviation around the median. J Exp Soc Psychol. 2013 July 1;49(4):764–6.

6.	Puonti O, Iglesias JE, Van Leemput K. Fast and sequence-adaptive whole-brain segmentation using parametric Bayesian modeling. NeuroImage. 2016 Dec 1;143:235–49.

7.	Fortin JP, Sweeney EM, Muschelli J, Crainiceanu CM, Shinohara RT. Removing inter-subject technical variability in magnetic resonance imaging studies. NeuroImage. 2016 May 15;132:198–212.

8.	Johnson WE, Li C, Rabinovic A. Adjusting batch effects in microarray expression data using empirical Bayes methods. Biostatistics. 2007 Jan 1;8(1):118–27.

9.	Fortin JP, Cullen N, Sheline YI, Taylor WD, Aselcioglu I, Cook PA, et al. Harmonization of cortical thickness measurements across scanners and sites. NeuroImage. 2018 Feb 15;167:104–20.

10.	Desikan RS, Ségonne F, Fischl B, Quinn BT, Dickerson BC, Blacker D, et al. An automated labeling system for subdividing the human cerebral cortex on MRI scans into gyral based regions of interest. NeuroImage. 2006 July 1;31(3):968–80.

11.	R Core Team. R: A Language and Environment for Statistical Computing [Internet]. Vienna, Austria: R Foundation for Statistical Computing; 2020. Available from: https://www.R-project.org/

12.	Steiger JH. Tests for comparing elements of a correlation matrix. Psychol Bull. 1980;87(2):245–51.

13.	Barth C, Kelly S, Nerland S, Jahanshad N, Alloza C, Ambrogi S, et al. In vivo white matter microstructure in adolescents with early-onset psychosis: a multi-site mega-analysis. Mol Psychiatry. 2023 Mar;28(3):1159–69.

14.	Viechtbauer W. Conducting Meta-Analyses in R with the metafor Package. J Stat Softw. 2010 Aug 5;36:1–48.

15.	Thorlund K, Imberger G, Johnston BC, Walsh M, Awad T, Thabane L, et al. Evolution of Heterogeneity (I2) Estimates and Their 95% Confidence Intervals in Large Meta-Analyses. PLoS ONE. 2012 July 25;7(7):e39471.

16.	Castro-Fornieles J, Baeza I, de la Serna E, Gonzalez-Pinto A, Parellada M, Graell M, et al. Two-year diagnostic stability in early-onset first-episode psychosis. J Child Psychol Psychiatry. 2011;52(10):1089–98.

17.	Pelizza L, Plazzi E, Leuci E, Leucci AC, Quattrone E, Azzali S, et al. Diagnostic shift in adolescents with first episode psychosis: findings from the 2-year follow-up of the “Parma Early Psychosis” program. Soc Psychiatry Psychiatr Epidemiol. 2024 June 29;60(2):375.

18.	Fox MP, MacLehose RF, Lash TL. Misclassification. In: Fox MP, MacLehose RF, Lash TL, editors. Applying Quantitative Bias Analysis to Epidemiologic Data [Internet]. Cham: Springer International Publishing; 2021 [cited 2026 Feb 5]. p. 141–95. Available from: https://doi.org/10.1007/978-3-030-82673-4_6

19.	Bromet EJ, Kotov R, Fochtmann LJ, Carlson GA, Tanenberg-Karant M, Ruggero C, et al. Diagnostic Stability Revisited: Shifts during the Decade Following First-Admission for Psychosis. Am J Psychiatry. 2011 Nov;168(11):1186–94.

20.	Hollis C. Adult Outcomes of Child- and Adolescent-Onset Schizophrenia: Diagnostic Stability and Predictive Validity. Am J Psychiatry. 2000 Oct;157(10):1652–9.

21.	Fusar-Poli P, Cappucciati M, Rutigliano G, Heslin M, Stahl D, Brittenden Z, et al. Diagnostic Stability of ICD/DSM First Episode Psychosis Diagnoses: Meta-analysis. Schizophr Bull. 2016 Nov 1;42(6):1395–406.

22.	Jarbin H, von Knorring AL. Diagnostic stability in adolescent onset psychotic disorders. Eur Child Adolesc Psychiatry. 2003 Feb 1;12(1):15–22.

23.	Fraguas D, de Castro MJ, Medina O, Parellada M, Moreno D, Graell M, et al. Does Diagnostic Classification of Early-Onset Psychosis Change Over Follow-Up? Child Psychiatry Hum Dev. 2008 June 1;39(2):137–45.
